# Meta-analysis of mouse transcriptomic studies supports a context-dependent astrocyte reaction in acute CNS injury versus neurodegeneration

**DOI:** 10.1186/s12974-020-01898-y

**Published:** 2020-07-31

**Authors:** Sudeshna Das, Zhaozhi Li, Ayush Noori, Bradley T. Hyman, Alberto Serrano-Pozo

**Affiliations:** 1 MGH BioMedical Informatics Core (BMIC), Cambridge, MA 02139 USA; 2 Department of Neurology, Massachusetts General Hospital, Boston, MA 02114 USA; 3 Massachusetts Alzheimer’s Disease Research Center, 114 16th street, Suite 2012, Charlestown, MA 02129 USA; 4 Harvard Medical School, Boston, MA 02116 USA

**Keywords:** Acute CNS injury, Astrocyte reaction, Meta-analysis, Neurodegenerative diseases, Transcriptomics

## Abstract

**Background:**

Neuronal damage in acute CNS injuries and chronic neurodegenerative diseases is invariably accompanied by an astrocyte reaction in both mice and humans. However, whether and how the nature of the CNS insult—acute versus chronic—influences the astrocyte response, and whether astrocyte transcriptomic changes in these mouse models faithfully recapitulate the astrocyte reaction in human diseases remains to be elucidated. We hypothesized that astrocytes set off different transcriptomic programs in response to acute versus chronic insults, besides a shared “pan-injury” signature common to both types of conditions, and investigated the presence of these mouse astrocyte signatures in transcriptomic studies from human neurodegenerative diseases.

**Methods:**

We performed a meta-analysis of 15 published astrocyte transcriptomic datasets from mouse models of acute injury (*n* = 6) and chronic neurodegeneration (*n* = 9) and identified pan-injury, acute, and chronic signatures, with both upregulated (UP) and downregulated (DOWN) genes. Next, we investigated these signatures in 7 transcriptomic datasets from various human neurodegenerative diseases.

**Results:**

In mouse models, the number of UP/DOWN genes per signature was 64/21 for pan-injury and 109/79 for acute injury, whereas only 13/27 for chronic neurodegeneration. The pan-injury-UP signature was represented by the classic cytoskeletal hallmarks of astrocyte reaction (*Gfap* and *Vim*), plus extracellular matrix (i.e., *Cd44*, *Lgals1, Lgals3, Timp1*), and immune response (i.e., *C3, Serping1, Fas, Stat1, Stat2, Stat3*). The acute injury-UP signature was enriched in protein synthesis and degradation (both ubiquitin-proteasome and autophagy systems), intracellular trafficking, and anti-oxidant defense genes, whereas the acute injury-DOWN signature included genes that regulate chromatin structure and transcriptional activity, many of which are transcriptional repressors. The chronic neurodegeneration-UP signature was further enriched in astrocyte-secreted extracellular matrix proteins (*Lama4*, *Cyr61*, *Thbs4*), while the DOWN signature included relevant genes such as *Agl* (glycogenolysis), *S1pr1* (immune modulation), and *Sod2* (anti-oxidant). Only the pan-injury-UP mouse signature was clearly present in some human neurodegenerative transcriptomic datasets.

**Conclusions:**

Acute and chronic CNS injuries lead to distinct astrocyte gene expression programs beyond their common astrocyte reaction signature. However, caution should be taken when extrapolating astrocyte transcriptomic findings from mouse models to human diseases.

## Background

Neuronal loss in central nervous system (CNS) acute injuries and chronic neurodegenerative diseases is invariably accompanied by an astrocyte reaction, which has been traditionally depicted by an increased glial fibrillary acidic protein (GFAP) immunoreactivity. However, thanks to the improvement of cell purification protocols and the development of transcriptomic methodologies (microarray and RNA-seq), the complexity and heterogeneity of this astrocyte reaction is emerging above and beyond this increased GFAP immunoreactivity. One area of uncertainty is whether there is any disease-specificity in astrocyte reaction or, conversely, this is just a non-specific response of astrocytes to any kind of neuronal injury. More specifically, whether reactive astrocytes could be neurotoxic in certain pathological conditions and neuroprotective in others is an area of active research. For example, it has been proposed that reactive astrocytes are neurotoxic (so called A1 astrocytes) in the lipopolysaccharide (LPS)-induced sepsis mouse model, but neuroprotective (termed A2 astrocytes) in the middle cerebral artery occlusion (MCAO) stroke mouse model [1, 2]. Another controversial question is whether astrocyte proliferation is a universal feature of astrocyte reaction. For example, astrocyte proliferation has been demonstrated in acute injury conditions such as traumatic brain injury (TBI) and stroke [3–5] but appears more limited in the context of chronic neurodegenerative diseases [5–9].

We hypothesized that astrocytes set off different transcriptomic programs in response to acute versus chronic disease, but that a common pan-injury signature underlies both types of conditions. To test this hypothesis, we took advantage of publicly available microarray and RNA-seq mouse astrocyte transcriptomic datasets from acute lesions [LPS-induced sepsis, MCAO stroke, spinal cord injury (SCI), and 1-methyl-4-phenyl-1,2,3,6-tetrahydropyridine (MPTP)] and chronic neurodegenerative disease models [amyotrophic lateral sclerosis (ALS) and Alzheimer’s disease (AD)], and applied bioinformatics tools [gene set enrichment analysis (GSEA) and meta-analysis]. Specifically, we asked whether (1) the previously reported A1, A2, and pan-reactive signatures can be extrapolated to conditions beyond LPS-induced sepsis and MCAO stroke; (2) distinct acute, chronic, and pan-injury (common to acute and chronic conditions) signatures can be identified; (3) reactive astrocytes in these conditions activate proliferative pathways as much as inflammatory cascades; and (4) astrocyte transcriptomic signatures obtained from mouse models correlate with changes in astrocyte gene expression levels in the corresponding human diseases.

## Methods

All analyses were conducted using R version 3.5.1 [10]

### Mouse gene expression analysis

Publicly available transcriptomic datasets of mouse astrocytes were identified either by using search terms in Gene Expression Omnibus (GEO) [11] or from relevant literature (Table 1). Microarray data were processed and normalized using RMA package in R. For RNA-seq datasets, we used fragments per kilobase of transcript per million mapped reads (FPKM) values whenever available; when only the raw sequencing fastq files were available, we used the *salmon* package to process and estimate transcript abundance [24]. Differentially expressed genes (DEGs) were identified using *limma* [25] and *voom+limma* [26] as genes with a statistically significant difference in expression level (*p* < 0.05) between the diseased (i.e., transgenic) and control (i.e., wild-type) mice.

**Table 1:** Summary of publicly available mouse astrocyte transcriptomic datasets used in this study

Label	Injury type	Comparison	N	Sex (*n* M/F)	Age	CNS region	Astrocyte isolation method	RNA method	Accession #	Ref.
LPS	Acute	LPS vs placebo	5/4	5M/4M	30–35 days	ctx+cc	FACS of Aldh1L1-eGFP+ cells	Microarray	GSE35338	[1]
MCAO-1	Acute	MCAO vs sham surgery	3/3	3M/3M	30–35 days	ctx+cc+hipp+str	FACS of Aldh1L1-eGFP+ cells	Microarray	GSE35338	[1]
MCAO-2	Acute	Transient MCAO ipsi- vs contra and control (no TMX)	3/3	NA	3–5 months	Hemibrain	Cx43-Cre-ERT/RiboTag	RNA-seq	GSE103783	[12]
SCI-1	Acute	SCI at T10 vs non-injured	4/4	4F/4F	2–4 months	Spinal cord	mGFAP-Cre-RiboTag	RNA-seq	GSE76097	[13]
SCI-2	Acute	SCI at T9 (hemi-and full transection vs non-injured)	9/3	9M/3M	12 weeks	Spinal cord	FACS of Aldh1L1-eGFP+ cells	RNA-seq	GSE96054	[14]
MPTP-24 h	Acute	MPTP vs vehicle (sac 24 h post-single i.p. inj.)	3/3	3F/3F	4–7 months	str	TRAP of Aldh1L1-eGFP-L10a	Microarray	10.17632/ktgcp4mtk2.1	[15]
SOD1-G93A-1	Chronic	G93A SOD1 vs wt	4/3	NA	90 days	Spinal cord	FACS Aldh1L1-eGFP/G93A SOD1	Microarray	GSE111031	[16]
SOD1-G93A-2	Chronic	G93A SOD1 vs wt	3/3	3M/3M	120 days	Spinal cord	LCM of Aldh1L1+ cells	Microarray	GSE69166	[17]
SOD1-G37R	Chronic	G37R SOD1 vs wt	4/6	4M/6M	8 months	Spinal cord	TRAP of Aldh1L1-eGFP-L10a	RNA-seq	GSE74724	[18]
APPPS1-1	Chronic	APPswePS1dE9 vs wt	4/4	NA	15–18 months	Whole ctx	FACS with Glt-1 Ab	Microarray	GSE74615	[19]
APPPS1-2	Chronic	APPswePS1dE9 vs wt	4/4	NA	15–18 months	Whole ctx	FACS of GFAP-GFP cells	Microarray	GSE74614	[20]
APPPS1-3	Chronic	APPswePS1dE9-GFP vs wt-GFP	4/7	4M/7M	9 months	CA1 hipp	FACS of eGFP+ cells	RNA-seq	GSE108520	[21]
PS2APP-1	Chronic	PS2APP vs wt	4/5	2M2F/1M4F	13 months	Whole brain	FACS with GFAP Ab	RNA-seq	GSE75431	[22]
PS2APP-2	Chronic	PS2APP vs wt	5/5	NA	11.5 months	Whole brain	FACS with GFAP Ab	RNA-seq	GSE129770	[23]
P301S-tau	Chronic	hMAPT-P301S tau vs wt	5/6	5M/6M	6 months	Whole ctx	FACS with GFAP Ab	RNA-seq	GSE129797	[23]

Abbreviations: *Ab* antibody, *Aldh1L1* aldehyde dehydrogenase 1 L1, *APP* amyloid-β precursor protein, *CA1* cornus ammonis 1, *cc* corpus callosum, *CNS* central nervous system, *ctx* cortex, *Cx43* connexin-43, *eGFP* enhanced green fluorescent protein, *FACS* fluorescence-activated cell sorting, *GFAP* glial fibrillary acidic protein, *Glt1* glutamate transporter 1, *i.p.* intra-peritoneal, *LCM* laser capture microdissection, *LPS* lipopolysaccharide, *MAPT* microtubule-associated protein tau, *MCAO* middle cerebral artery occlusion, *MPTP* 1-methyl-4-phenyl-1,2,3,6-tetrahydropyridine, *PS1/2* presenilin 1/2, *sac* sacrifice, *SCI* spinal cord injury, *SOD1* superoxide dismutase 1, *TRAP* translating ribosome affinity purification

### Meta-analysis of mouse transcriptomic studies

Meta-analyses were conducted using the *sumz* function in the metap package in R, which first converts the *P* values into *Z* scores, and then computes the composite *Z* score [27]. Square root of the sample sizes was used as weights in the *sumz* procedure to control for the number of samples in each study. The *p* values were corrected for multiple comparisons using a false discovery rate (FDR) < 5%. We conducted meta-analyses within acute injury and neurodegenerative mouse studies separately. We identified the pan-injury signature as those genes that met two criteria: (1) had a statistically significant multiple-comparison-corrected meta *p* value (meta *p* < 0.05) and (2) had a statistically significant (*p* < 0.05) differential expression in at least 33% of both acute injury and neurodegenerative mouse datasets. The chronic neurodegeneration-specific signature was defined as those genes with a statistically significant adjusted meta *p* value (meta *p* < 0.05) in the neurodegenerative meta-analysis, which had a statistically significant differential expression (*p* < 0.05) in 33% or more neurodegenerative studies but none of the acute injury datasets. Similarly, the acute injury-specific signature was defined as those genes with a statistically significant adjusted meta *p* value (meta *p* < 0.05) in the acute injury meta-analysis, which had a statistically significant differential expression (*p* < 0.05) in 33% or more acute injury studies but none of the chronic neurodegeneration datasets. DEGs were considered upregulated (UP) if the logarithm of the fold change (logFC) was > 0 and downregulated (DOWN) if the logFC was < 0. Although some of the datasets analyzed had some contamination from microglial genes [17, 18, 21], this analytic approach minimized the probability of including these microglial genes from a single dataset in the final meta-analytic signatures. Moreover, we confirmed the expression of the resulting genes by astrocytes in a previously published RNA-seq study of cell subpopulations isolated from the mouse brain [28].

### Gene set enrichment analysis (GSEA)

To identify signaling pathways, we searched for the terms “KAPPA”, “NFAT”, “MAPK”, “JAK/STAT”, “WNT/BETA-CATENIN”, and “SONIC HEDGEHOG” within the KEGG, REACTOME, BIOCARTA, PID, and GO pathways compiled in the MSigDB version 6.2 [29] and obtained a total of 86 gene sets. Next, we performed gene set enrichment analysis (GSEA) [30, 31] to determine the enrichment of each of these gene sets in each of the 15 mouse astrocyte transcriptomic datasets and generated a heatmap with the normalized enrichment scores (NES) and another heatmap with the − log_10_ of the *p* values. Next, we conducted a meta-analysis of the resulting GSEA *p* values and ranked the gene sets in descending adjusted meta *p* value, representing the relative relevance of these signaling pathways in the astrocytes from each of the mouse models.

### Validation of mouse astrocyte signatures in human transcriptomic datasets

To validate the meta-analytic mouse transcriptomic signatures, we investigated the differential expression of each of the signature genes in human microarray and RNA-seq bulk tissue and astrocyte-specific datasets from AD, Parkinson’s disease (PD), and ALS. Table 2 depicts the human datasets used in these analyses, with sample size, CNS region of interest, transcriptomic method, accession number, and reference. The AD datasets were analyzed by comparing individuals with Braak NFT V/VI (AD) versus Braak NFT 0/I/II (controls), as described previously [37]. The differential expression of diseased versus control individuals for the PD and ALS datasets was analyzed using *limma*. Violin plots were generated using ggplot2 version 3.2.1 [38] to represent the logFC of each signature gene in diseased versus control individuals.

**Table 2:** Summary of publicly available human neurodegenerative transcriptomic datasets used in this study

Label	Comparison	N	Sample type	RNA method	CNS region	Accession #	Ref.
**AD-DLPC**	AD (Braak V/VI) vs CTRL (Braak 0/I/II)	140/112	Bulk tissue	RNA-seq	Dorsolateral prefrontal cortex	syn3505720	[32]
**AD-PHG**	AD (Braak V/VI) vs CTRL (Braak 0/I/II)	62/79	Bulk tissue	RNA-seq	Parahippocampal gyrus	syn5898488	[33]
**AD-astro1**	AD (Braak V/VI) vs CTRL (Braak 0/I/II)	6/6	LCM of GFAP+ astrocytes	Microarray	Lateral temporal cortex	GDS4135	[34]
**AD-astro2**	AD (Braak V/VI) vs CTRL (Braak 0/I/II)	7/12	FACS with GFAP Ab	RNA-seq	Superior frontal gyrus	GSE125050	Friedman, Hansen (unpublished)
**PD-SN**	PD vs CTRL	3/3	Bulk tissue	Microarray	Substantia nigra	GSE54282	[35]
**PD-Str**	PD vs CTRL	6/6	Bulk tissue	Microarray	Striatum	GSE54282	[35]
**ALS-SC**	SALS vs CTRL	5/4	Bulk tissue	Microarray	Spinal cord gray matter	GSE833	[36]

Abbreviations: *Ab* antibody, *AD* Alzheimer’s disease, *ALS* amyotrophic lateral sclerosis, *CNS* central nervous system, *CTRL* control, *DLPC* dorsolateral prefrontal cortex, *FACS* fluorescence-activated cell sorting, *GFAP* glial fibrillary acidic protein, *LCM* laser capture microdissection, *PD* Parkinson’s disease, *PHG* parahippocampal gyrus, *SALS* sporadic ALS, *SC* spinal cord, *SN* substantia nigra, *Str* striatum

## Results

### Compilation of acute injury and neurodegenerative astrocyte transcriptomic datasets

A total of 15 transcriptomic datasets from astrocytes isolated from mouse models were obtained from GEO. Dataset category (acute injury versus neurodegeneration), mouse model, mouse age and sex, CNS region, method of astrocyte isolation, GEO accession number, and literature reference for these datasets can be found in Table 1. Mouse acute injury datasets (*n* = 6) included LPS-induced sepsis (*n* = 1) [1], MCAO stroke (*n* = 2) [1, 12], SCI (*n* = 2) [13, 14], and acute toxic parkinsonism (MPTP, *n* = 1) [15]. For one of the stroke studies [1], only the dataset from 3 days after MCAO was analyzed, because gene expression changes were maximum 3 days and attenuated by 7 days after MCAO surgery. The MPTP dataset [15] was classified as acute injury rather than chronic neurodegeneration because the authors examined the changes in astrocyte transcriptome after an acute neurotoxic injury to the substantia nigra dopaminergic neurons (i.e., 12 h, 24 h, and 48 h after a single MPTP intra-peritoneal injection), rather than after chronic MPTP administration through a subcutaneous osmotic pump [39]. Since the three acute MPTP time points are not independent datasets, only the 24 h dataset was used for the meta-analysis [15]. Mouse neurodegenerative datasets (*n* = 9) included ALS (various SOD1 mutants, *n* = 3) [16–18] and AD models of both brain β-amyloidosis (*n* = 5) (APPswe/PSEN1deltaE9 *n* = 3 and PS2APP *n* = 2) [19–23], and tauopathy (P301S-MAPT, *n* = 1) [23]. Whenever more than one age group was available, only the older age group was analyzed.

### A1 and A2 astrocyte transcriptomic signatures are not CNS disease-specific

**Fig. 1:**
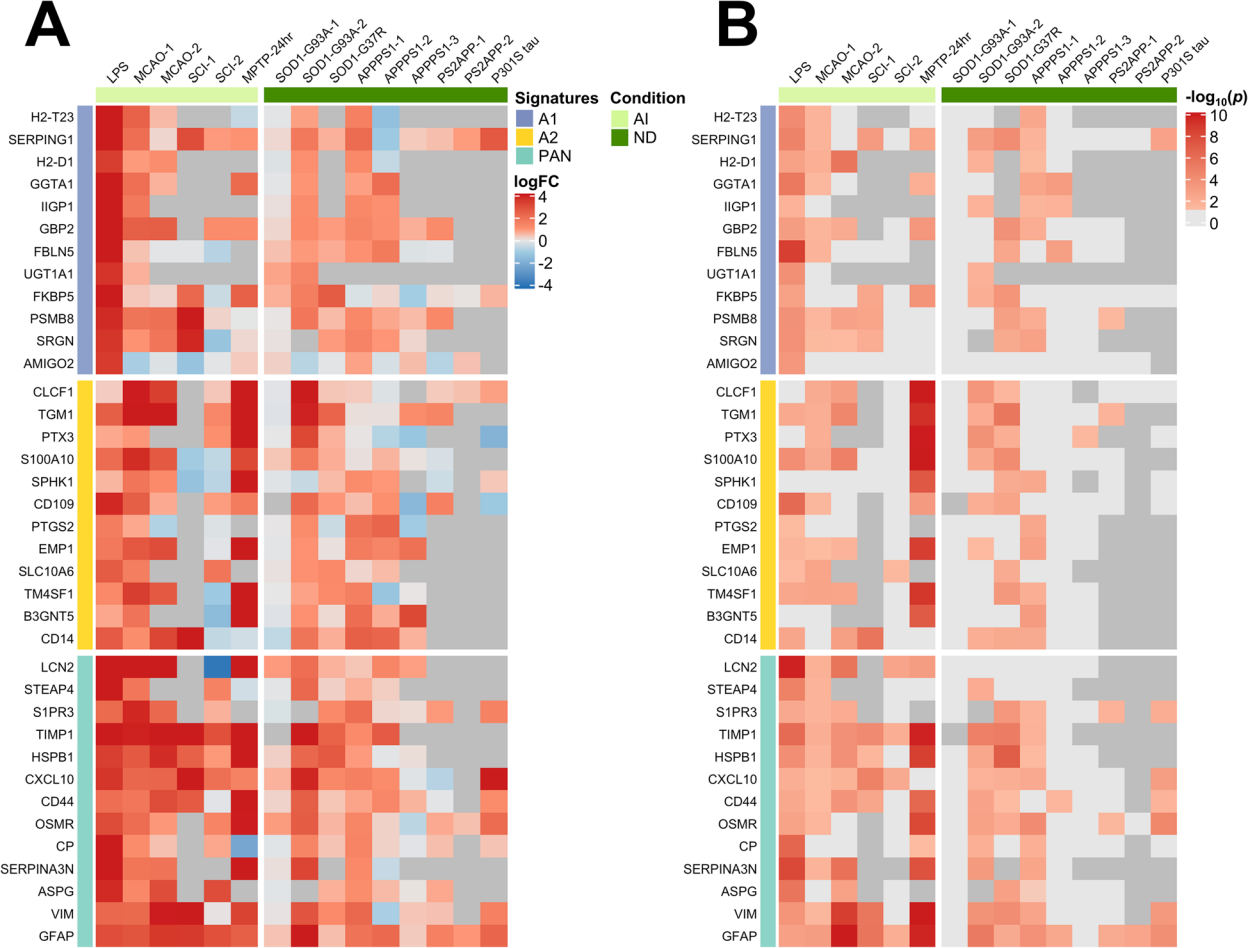
Purported neurotoxic, neuroprotective, and pan-reactive astrocyte transcriptomic signatures in acute injury and chronic neurodegenerative mouse models. Heatmaps illustrate the log_2_(fold-change) (**a**) and − log_10_(*p* values) (**b**) of the neurotoxic (A1), neuroprotective (A2), and pan-reactive (PAN) gene cassettes as defined by Liddelow et al. [2]. Dark gray in (**a**) and (**b**) means that the transcript was not detected or was detected at extremely low levels. Light gray transcripts in (**b**) were not statistically significant. Note that all three signatures are significantly upregulated in astrocytes from chronic neurodegeneration (ND) and, specially, acute injury (AI) mouse models

Recently, an A1 neurotoxic signature and an A2 neuroprotective signature have been defined based on the DEGs from two acute injury mouse models: LPS-induced sepsis and MCAO stroke, respectively, with a third “pan-reactive” signature representing the common overlap between sepsis- and stroke-induced transcriptomic changes [1, 2]. Moreover, it has been suggested that, in neurodegenerative diseases, astrocytes also upregulate the A1 neurotoxic signature and downregulate the A2-neuroprotective signature [2]. To test the hypothesis that these A1, A2, and pan-reactive signatures can be extrapolated to other acute injuries as well as neurodegenerative diseases, we examined the differential expression of the genes comprising these signatures in the 15 mouse datasets described above. The heatmaps in Fig. 1 show the fold change of the expression of the genes comprising these three signatures and the *p* values of these comparisons. Surprisingly, overall the A1, A2, and pan-reactive genes were upregulated in astrocytes across most acute injury and neurodegeneration mouse models. Only MPTP appeared to induce a differential response, with an upregulation of the A2 and pan-reactive signatures and a downregulation of the A1 signature. Of note, astrocytes from acute injury mouse models tended to exhibit higher level of expression of all three gene cassettes than those from neurodegeneration mouse models. Overall, these analyses demonstrate that the previously described A1 and A2 signatures do not discern between CNS conditions and, in fact, are not mutually exclusive, but part of a broader pan-injury program that is common to both acute injury and chronic neurodegenerative responses.

### Meta-analysis of mouse astrocyte transcriptomic studies reveals distinct signatures of astrocyte reaction in acute CNS injuries versus chronic neurodegenerative diseases

**Fig. 2:**
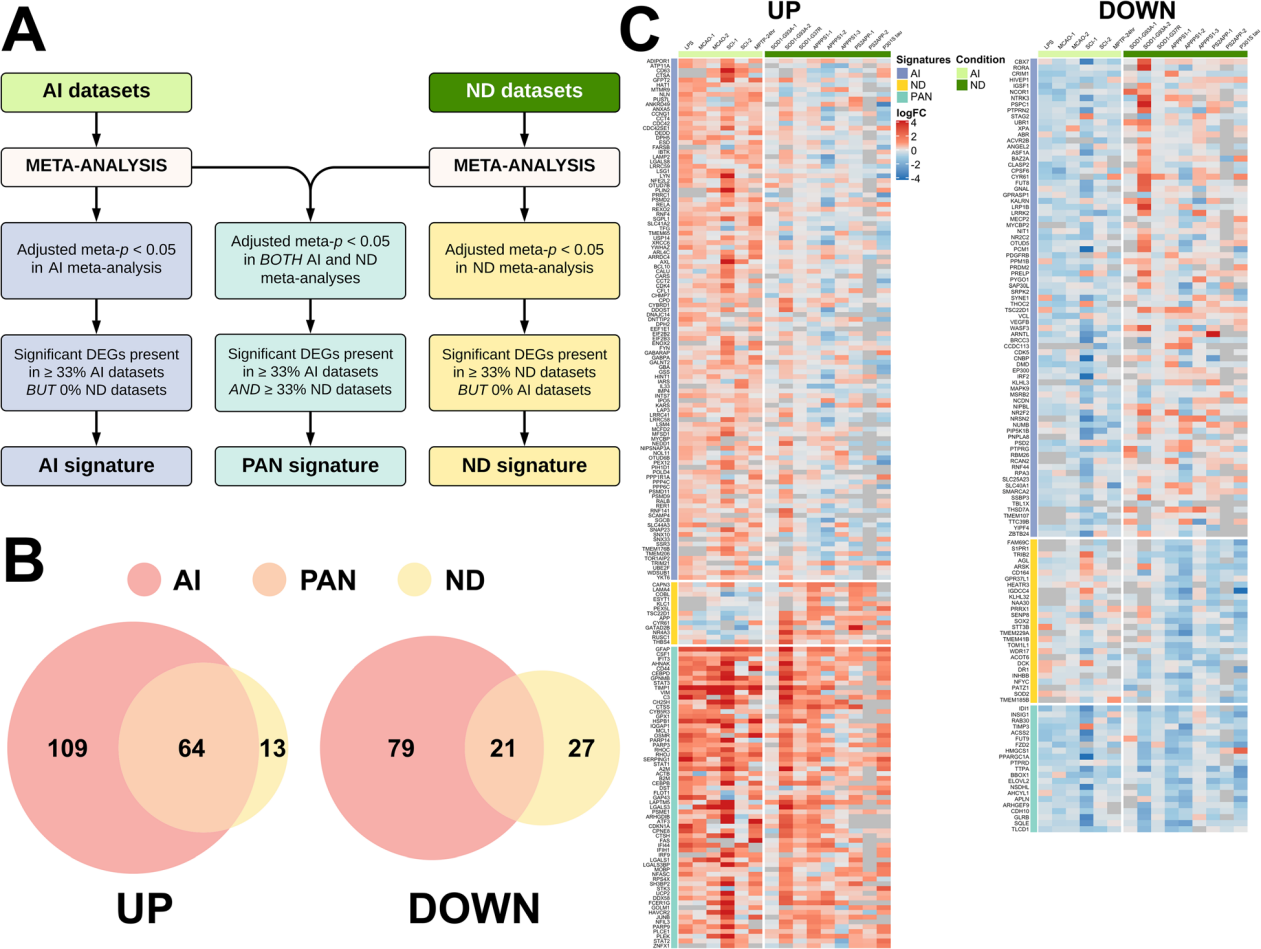
Meta-analysis shows distinct acute injury and chronic neurodegeneration signatures, and a common pan-injury signature. **a** Flow-chart shows the criteria used to develop acute injury (AI), chronic neurodegenerative diseases (ND), and pan-injury (PAN) signatures. **b** Venn diagrams show the number of upregulated (UP) and downregulated (DOWN) genes for each of the signatures. **c** Heatmaps depict log_2_(fold change) of upregulated (UP) and downregulated (DOWN) genes comprising the three signatures

Since the previously defined A1 and A2 signatures did not discriminate between injuries of very different nature, we next sought to describe new acute, chronic, and pan-injury astrocyte transcriptomic signatures. To this end, we conducted a meta-analysis of the DEGs, both upregulated (UP) and downregulated (DOWN), from the mouse acute and chronic datasets separately (Fig. 2a). We defined the pan-injury signature as genes with a statistically significant multiple-comparison-corrected meta-analysis *p* value (*p* < 0.05) in both acute and chronic datasets and which, as an additional filter, also reached statistical significance in at least 33% of both acute and chronic datasets (see the meta *p* value of each signature gene and the number of acute and chronic datasets in which it was significant in Supplemental Table 1). The chronic neurodegeneration-specific signature was defined as DEGs that had an adjusted meta *p* value < 0.05 in the meta-analysis of chronic neurodegeneration datasets, were present in 33% or more of the neurodegeneration datasets, and absent in all acute injury datasets. Similarly, the acute injury-specific signature was defined by DEGs that had an adjusted meta *p* value < 0.05 in the meta-analysis of acute injury datasets, were present in 33% or more of all the acute injury datasets, and absent in all chronic neurodegeneration datasets.

Using these criteria, we obtained an acute injury-specific signature with 109 upregulated and 79 downregulated genes; a chronic neurodegeneration-specific signature with 13 upregulated and 27 downregulated genes, and a pan-injury signature with 64 upregulated genes and 21 downregulated genes. The Venn diagrams in Fig. 2b and the heatmaps in Fig. 2c and Supplemental Figure 1 depict the results of the meta-analysis for the UP and DOWN signatures, respectively. To characterize these signatures, we next interrogated curated databases such as Gene Ontology (GO biological processes, cellular components, and molecular functions) and Kyoto Encyclopedia of Genes and Genomes (KEGG) and generated heatmaps of relevant gene cassettes.

### Pan-injury astrocyte transcriptomic signature

**Fig. 3:**
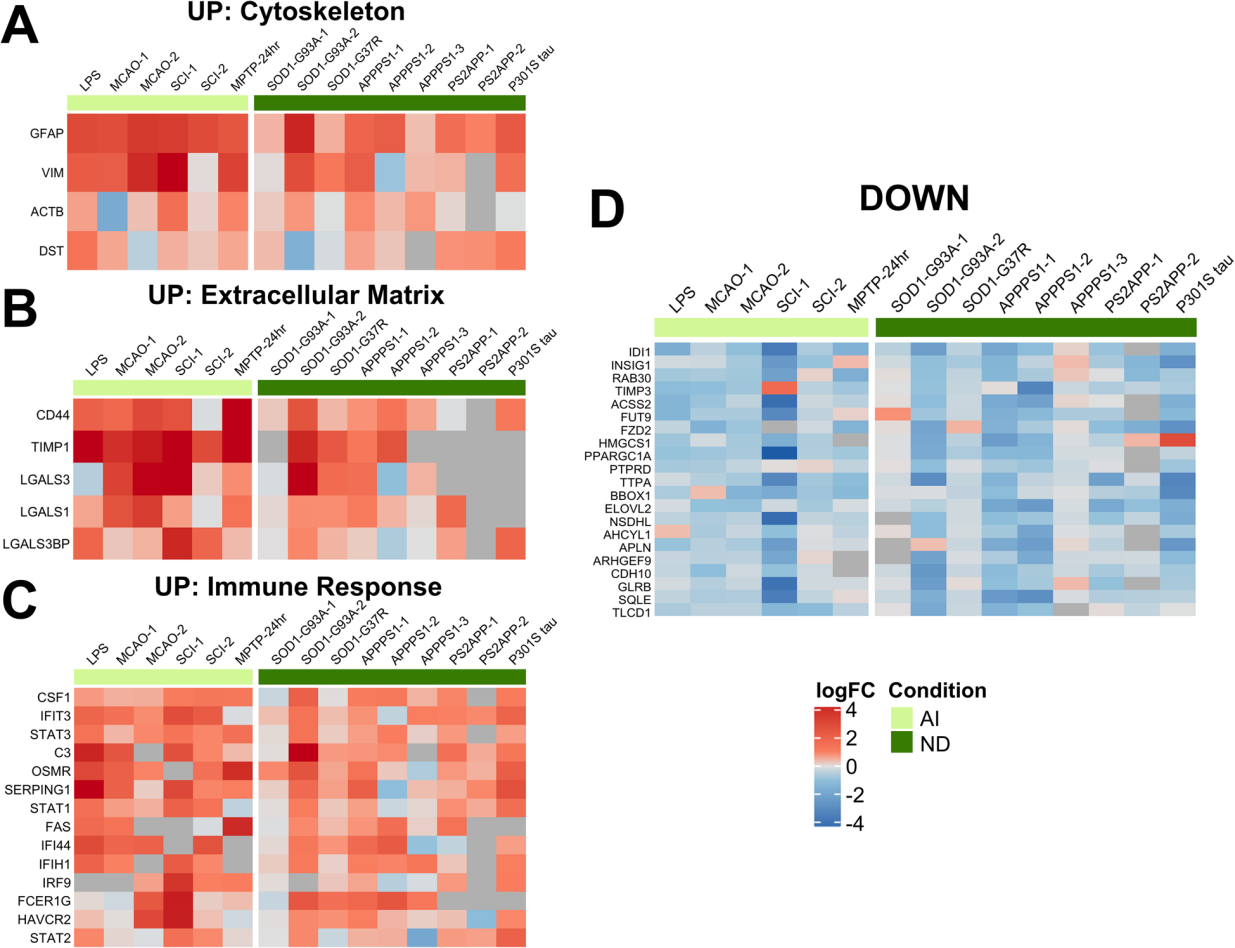
Pan-injury astrocyte gene expression signature. Heatmaps depict log_2_(fold change) of upregulated (**a**–**c**) and downregulated (**d**) genes in both acute injury (AI) and chronic neurodegeneration (ND). The pan-injury (PAN) signature consists of upregulation of cytoskeleton (i.e., *Gfap*, *Vim*) (**a**), extracellular matrix (i.e., *Cd44*, *Timp1*) (**b**), and immune response (i.e., *C3,* interferon signaling, STAT pathway) (**c**), and downregulation of lipid metabolism (i.e., *Hmgcs1*, *Sqle*) among other genes (**d**)

The pan-injury-UP signature corresponded to biological processes such as cytokine receptor and interferon signaling and to cellular components such as secretory vesicles, cell surface, and extracellular matrix. Specifically, this signature was represented by genes related to the cytoskeleton (*Actb, Dst*, *Gfap, Vim*), extracellular matrix (*Cd44*, *Lgals1*, *Lgals3*, *Lgals3bp*, *Timp1*), and immune response (*C3*, *Csf1*, *Fas*, *Fcer1g*, *Havcr2*, *Ifi44*, *Ifih1*, *Ifit3*, *Irf9, Osmr, Serping1*, *Stat1*, *Stat2*, *Stat3*), whereas the pan-injury-DOWN signature was comprised of genes with very diverse functions, although a lipid metabolism gene cassette (*Acss2*, *Bbox1*, *Elovl2*, *Fzd2*, *Hmgcs1*, *Sqle*) could be identified (Fig. 3).

### Acute injury-specific astrocyte transcriptomic signature

**Fig. 4:**
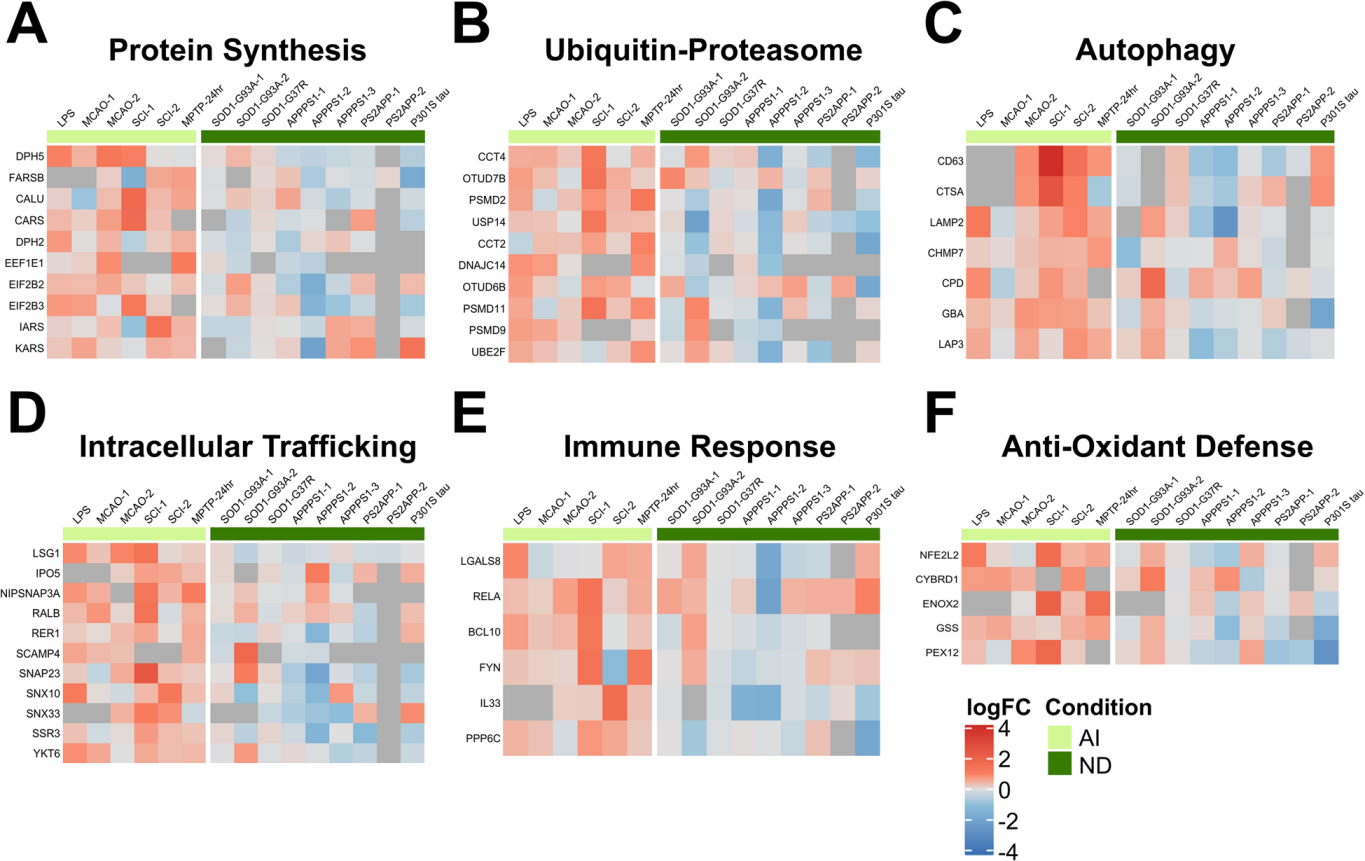
Astrocyte gene expression signature upregulated in acute injury. Heatmaps depict log_2_(fold change) of acute injury-specific upregulated genes. The upregulated acute injury signature consists of protein translation (i.e., elongation machinery and aminoacyl tRNA synthetases) (**a**), ubiquitin-proteasome system (i.e., proteasome subunits and chaperones) (**b**), autophagy system (i.e., lysosomal enzymes such as cathepsin A and glucocerebrosidase, and lysosomal associated membrane protein 2) (**c**), intracellular trafficking (i.e., ER-Golgi) (**d**), immune response (i.e., *Rela*, *Il33*) (**e**), and anti-oxidant defenses (i.e., *Nfe2l2*, *Gss*)

**Fig. 5:**
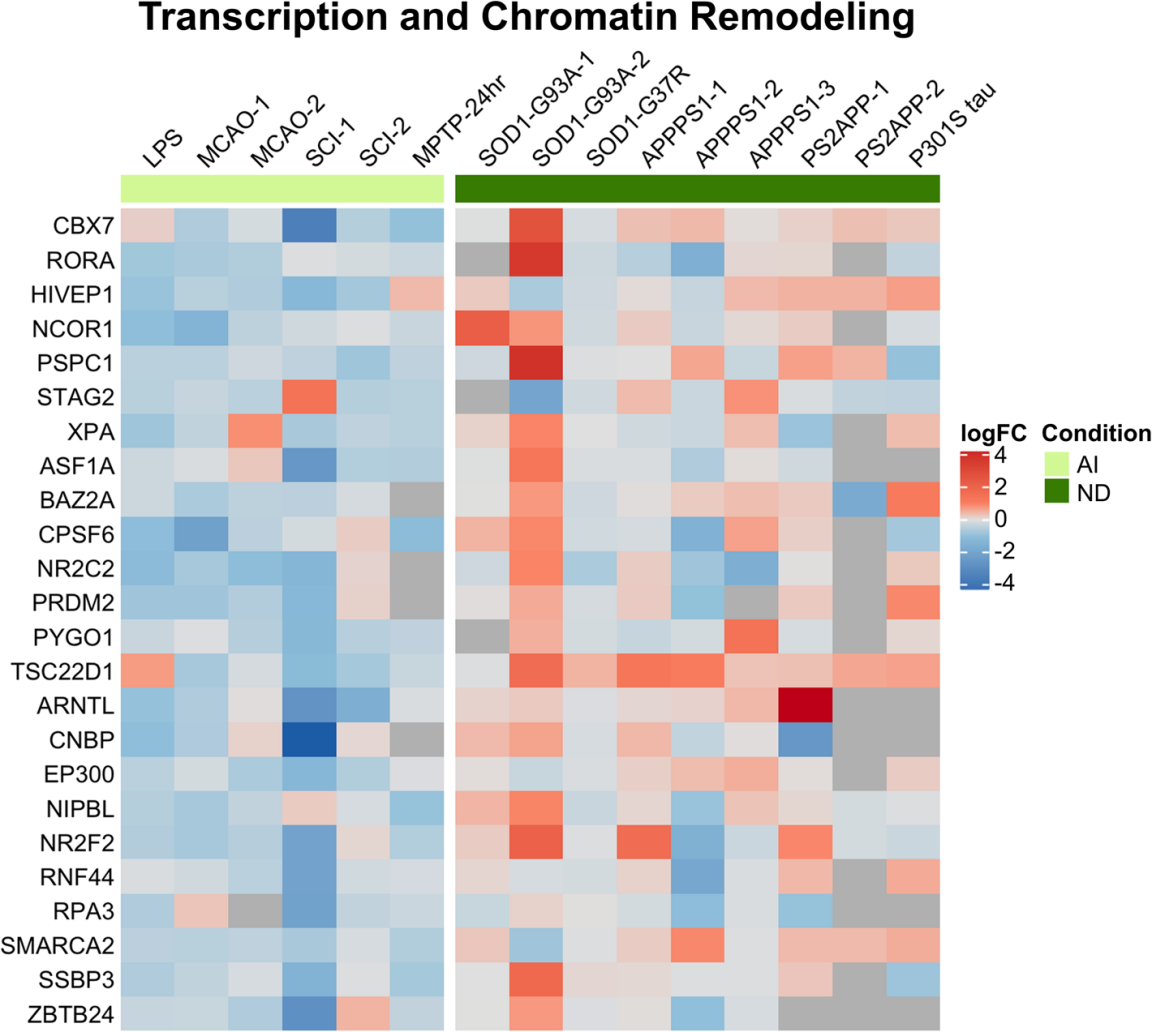
Astrocyte gene expression signature downregulated in acute injury. Downregulated genes in acute injury were mainly related to chromatin remodeling and organization and transcription, including many transcriptional repressors

The acute injury-UP signature corresponded to biological processes related to protein synthesis and degradation (proteasome and autophagy), response to cytokine and extracellular stimulus, and innate immune system, and to cell components such as endoplasmic reticulum, Golgi apparatus, and secretory granules and vesicles. Specifically, this signature includes gene cassettes related to protein synthesis (*Calu*, *Cars*, *Dph2*, *Dph5*, *Eef1e1*, *Eif2b2*, *Eif2b3, Farsb*, *Iars*, *Kars*), ER-Golgi trafficking (*Ipo5*, *Lsg1*, *Nipsnap3a*, *Ralb*, *Rer1*, *Scamp4*, *Snap23*, *Snx10*, *Snx33*, *Ssr3*, *Ykt6*), the ubiquitin-proteasome system (*Cct2*, *Cct4*, *Dnajc14*, *Otud6b*, *Otud7b*, *Psmd2*, *Psmd9*, *Psmd11*, *Ube2f*, *Usp14*), autophagy (*Chmp7, Cpd*, *Ctsa*, *Cd63*, *Gba*, *Lamp2*, *Lap3*), immune response (*Bcl10*, *Fyn*, *Rela*, *Lgals8*, *Ppp6c*), and anti-oxidant defense (*Cybrd1*, *Enox2*, *Gss*, *Nfe2l2*, *Pex12*) (Fig. 4). By contrast, the acute injury-DOWN signature was related to transcription and chromatin remodeling processes, localized to chromatin and cell nucleus, and was mainly comprised of genes that regulate chromatin structure and transcriptional activity (*Arntl*, *Asf1a*, *Baz2a*, *Cbx7*, *Cnbp*, *Cpsf6*, *Ep300*, *Hivep1*, *Ncor1*, *Nipbl, Nr2c2*, *Nr2f2*, *Prdm2*, *Pspc1, Pygo1*, *Rnf44*, *Rora*, *Rpa3*, *Smarca2*, *Ssbp3*, *Stag2*, *Tsc22d1*, *Xpa*, and *Zbtb24*) (Fig. 5). Of note, many of these transcripts encode transcriptional repressors (i.e., *Baz2a*, *Cbx7*, *Cnbp*, *Ncor1*, *Zbtb24*), therefore their downregulation would lead to an enhanced transcriptional activity in the astrocytes. Other downregulated gene cassettes of interest in this signature included immune response (*Igsf1*, *Irf2*, *Mapk9*, *Rcan2*) and trophic factors (*Acvr2b*, *Ntrk3*, *Pdgf3rb*, *Vegfb*).

### Chronic neurodegeneration-specific astrocyte transcriptomic signature

**Fig. 6:**
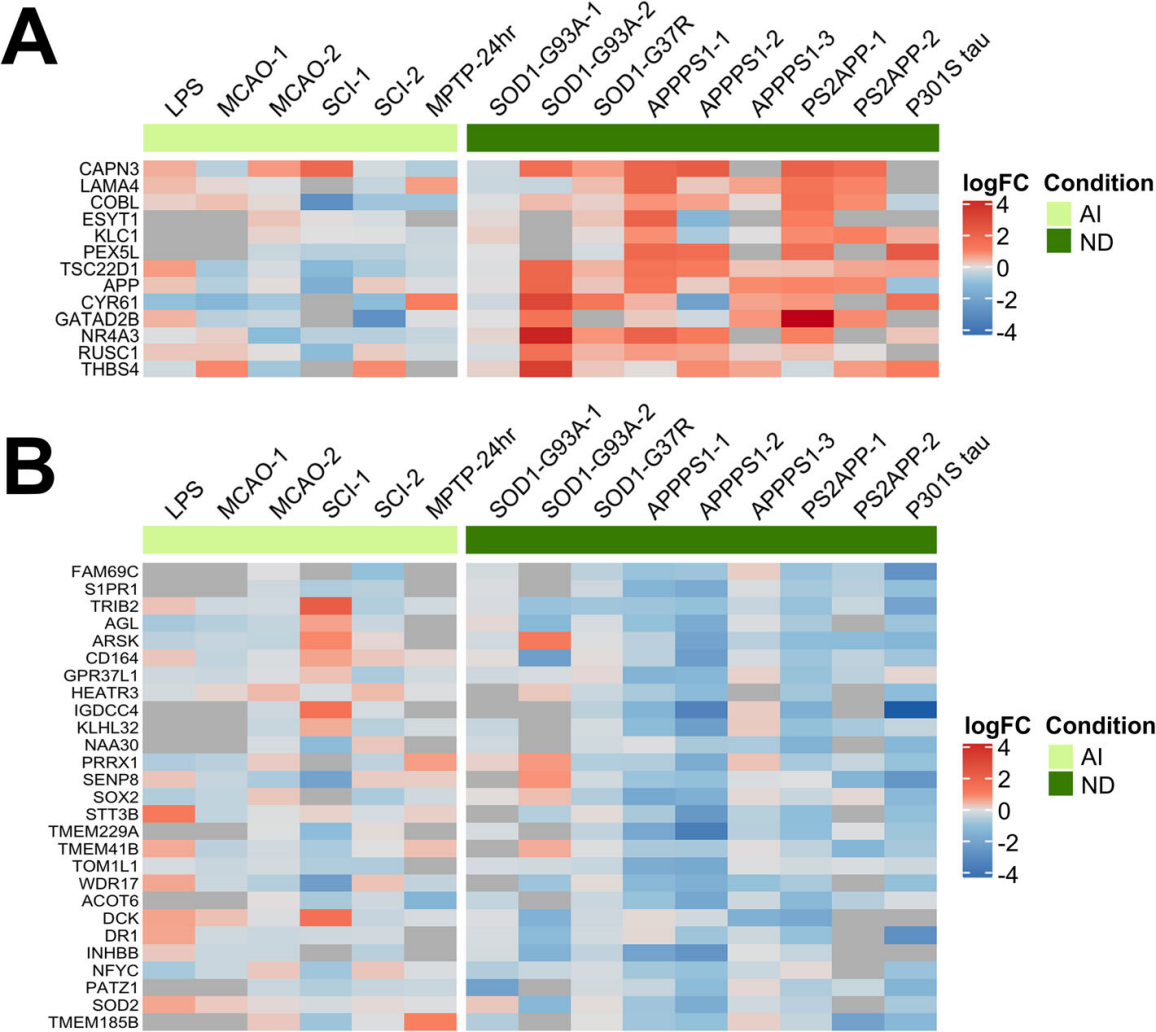
Astrocyte gene expression signature in chronic neurodegeneration. The chronic neurodegeneration signature is characterized by upregulation (**a**) of amyloid precursor protein, calpain-3, and secreted matricellular proteins (i.e., *Cyr61*, *Lama4*, *Thbs4*), and downregulation (**b**) of metabolic processes (i.e., *Acot6*, *Agl*, *Naa30*)

The chronic neurodegeneration-specific signature was notably the smallest in number of genes, and these turned out to be functionally very heterogeneous. The chronic neurodegeneration-UP signature (*n* = 13) included three genes encoding extracellular matrix proteins that are known to be secreted by astrocytes (*Cyr61*, *Lama4*, and *Thbs4*)*.* Other chronic neurodegeneration-UP genes were *App*, *Capn3*, *Cobl*, *Esyt1*, *Gatad2b*, *Klc1*, *Nr4a3, Pex5l*, *Rusc1*, and *Tsc22d1* (Fig. 6a). The chronic neurodegeneration-DOWN signature (*n* = 27) included a metabolic gene cassette (*Acot6*, *Agl*, *Arsk*, *Naa30*, *Stt3b*) and a transcription/chromatin remodeling gene cassette (*Dr1, Nfyc, Patz1*, *Prrx1*) together with relevant genes such as *Cd164*, *Heatr3*, *S1pr1*, and *Sod2* (Fig. 6b).

### Astrocyte reaction involves upregulation of NFκB, MAPK, JAK-STAT, and CaN-NFAT signaling pathways, but not Wnt/β-Catenin and Sonic hedgehog proliferative pathways

We next investigated which molecular signaling pathways are involved in astrocyte reaction in each condition and whether astrocyte proliferation is part of astrocyte reaction. Specifically, we hypothesized that acute CNS injuries would trigger astrocyte proliferation to create a scar and limit neuronal damage, whereas this proliferative potential of astrocytes could be exhausted in chronic neurodegenerative diseases. A literature review revealed four main signaling pathways involved in astrocyte reaction: nuclear factor kappa B (NFκB) [40], calcineurin-nuclear factor activating of T-cells (CaN-NFAT) [41–44], Janus Kinases/Signal Transducer and Activator of Transcription (JAK/STAT) [21, 45–47], and mitogen-activated protein kinase (MAPK) [34, 48–51], and two main pathways involved in astrocyte proliferation: Wnt/β-catenin [52, 53] and Sonic hedgehog [5, 54].

**Fig. 7:**
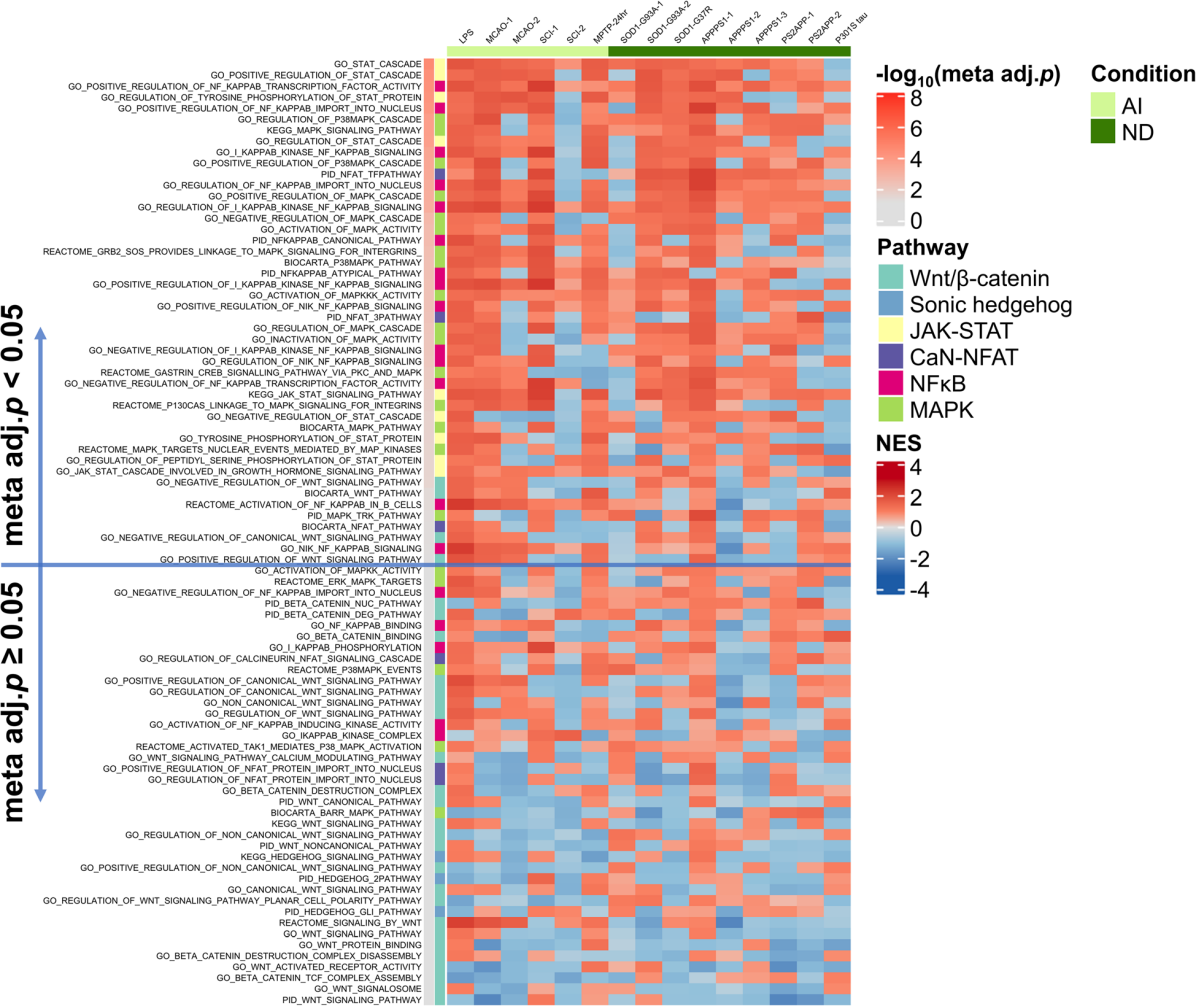
Inflammatory but not proliferation signaling pathways are upregulated in acute injury and chronic neurodegeneration. Heatmap shows the normalized enrichment score (NES) of pro-inflammatory (NFκB, calcineurin/NFAT, MAPK, and JAK/STAT) and proliferative (Wnt/β-catenin and sonic hedgehog) gene sets in the astrocyte transcriptomic datasets analyzed. The gene sets are color-coded by pathway and ordered by descending meta-analytic *p* value [− log10(Meta adj. *p*)]. Note that NFκB, MAPK, and JAK/STAT gene sets are more significantly upregulated than Wnt/β-catenin and sonic hedgehog

To investigate whether these pathways are upregulated in reactive astrocytes in a context-dependent fashion, we performed GSEA [30] with the gene sets comprising these six pathways (see the “Methods” section) on the 6 acute injury and 9 chronic neurodegeneration astrocyte transcriptomic datasets, followed by a meta-analysis of the resulting GSEA *p* values. We identified a total of 86 gene sets related to one of these six signaling pathways with the following distribution: Wnt/β-catenin *n* = 28, MAPK *n* = 21, NFκB *n* = 19, JAK/STAT *n* = 9, NFAT *n* = 6, and Sonic hedgehog *n* = 3. Figure 7 shows a heatmap with the NES resulting from the GSEA ranked by descending adjusted meta-analytic *p* value, whereas Supplemental Figure 2 heatmap depicts the − log_10_(*p* values) from this GSEA. The NFκB, JAK/STAT, and MAPK signaling pathways encompassed the top gene sets and were upregulated in most datasets from both acute injury and chronic neurodegeneration categories. By contrast, the Wnt/β-catenin and Sonic hedgehog gene sets were predominantly downregulated and did not reach the meta-analytic statistical significance in most instances. Of note, the astrocyte pan-injury signature described above included *Stat1*, *Stat2*, and *Stat3*, whereas the acute injury-UP signature included *Rela*, which encodes for the effector subunit of NFκB p65, and the acute injury-DOWN signature included *Rcan2*, which is the main repressor of Ca-N/NFAT signaling, suggesting that the JAK/STAT signaling pathway is active in all astrocyte reactions, whereas NFκB and NFAT signaling pathways are primarily turned on in response to acute injuries.

### Validation of mouse astrocyte signatures in human neurodegenerative diseases

**Fig. 8:**
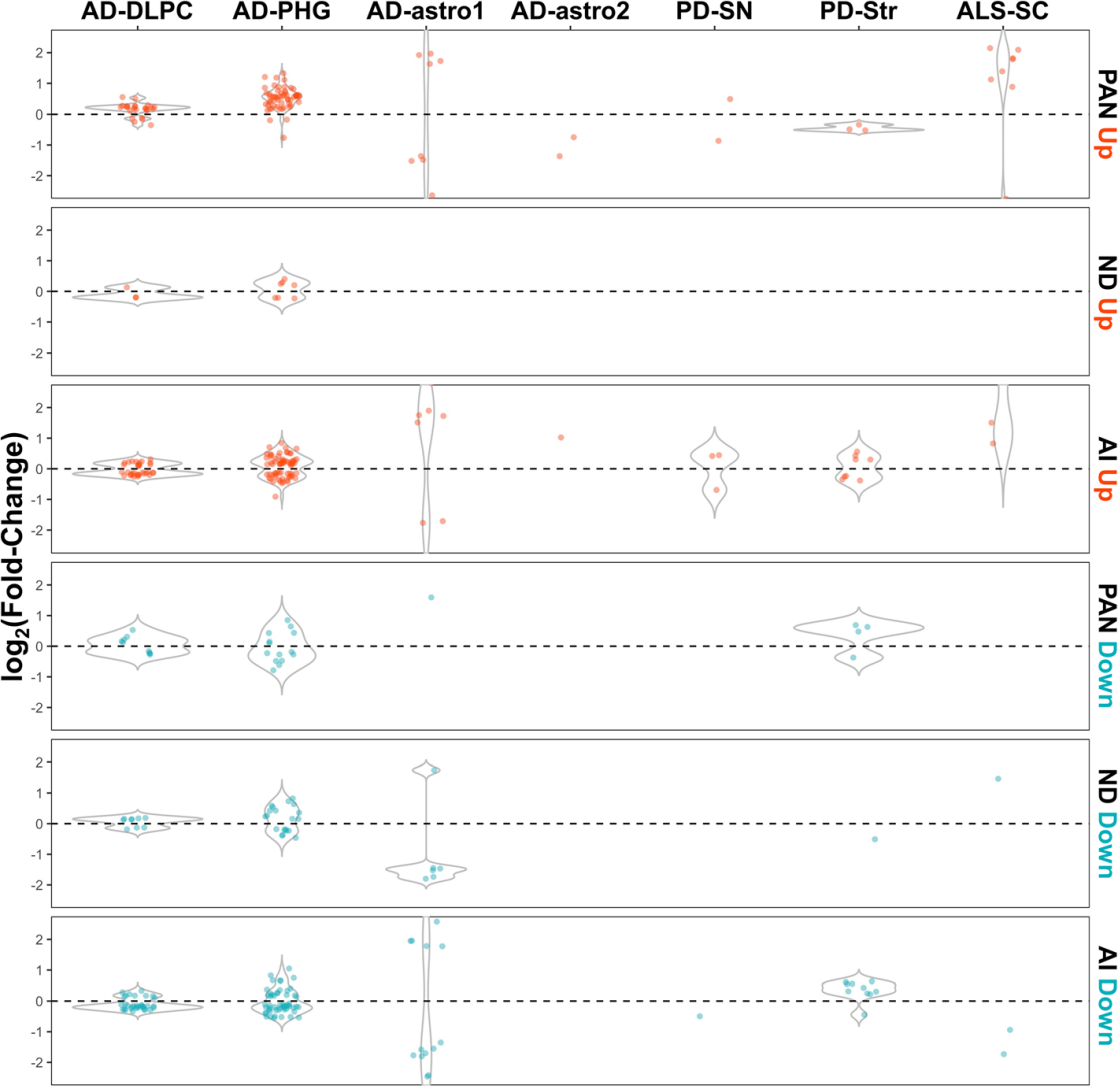
Validation of mouse astrocyte transcriptomic signatures in human neurodegenerative transcriptomic datasets. Violin plots represent the logFC of statistically significant DEGs (*p* < 0.05) from the acute injury (AI), chronic neurodegenerative (ND), and pan-injury (PAN) mouse astrocyte signatures in diseased versus control human subjects. Note that only the two AD bulk tissue datasets (AD-DLPC and AD-PHG) showed some concordance with the pan-injury-UP mouse astrocyte signatures, but not with any of the others. Abbreviations: AD-DLPC, AD dorsolateral prefrontal cortex from ROSMAP; AD-PHG, AD parahippocampal gyrus from MSBB; AD-astro1, GFAP+ astrocytes laser capture microdissected from lateral temporal cortex; AD-astro2, GFAP+ astrocytes sorted from superior frontal cortex; ALS-SC, amyotrophic lateral sclerosis spinal cord gray matter; PD-SN, Parkinson’s disease substantia nigra; PD-Str, Parkinson’s disease striatum

Lastly, we sought to validate the acute injury, chronic neurodegeneration, and pan-injury mouse astrocyte signatures in human brain transcriptomic (microarray and RNA-seq) datasets from neurodegenerative disease-relevant CNS regions. These included two large AD datasets, the Religious Orders Study and Memory and Aging Project (ROSMAP, dorsolateral prefrontal cortex) [32] and the Mount Sinai Brain Bank (MSBB, parahippocampal gyrus) [33], two PD datasets (substantia nigra and striatum) [35], and one sporadic ALS dataset (spinal cord gray matter) [36]. In addition to these bulk tissue transcriptomic datasets, we analyzed two astrocyte-specific AD datasets: one in which GFAP+ astrocytes were laser capture microdissected from lateral temporal cortex frozen sections [34], and another in which superior frontal gyrus astrocytes were labeled with a GFAP antibody and sorted from other cell types in the suspension through fluorescence-activated cell sorting (FACS) (Friedman and Hansen, unpublished). Statistically significant DEGs (*p* < 0.05) between diseased and control individuals are illustrated with violin plots in Fig. 8, whereas violin plots with all the mouse signature genes are shown in Supplemental Figure 3. Inspection of these violin plots indicates that most genes from the pan-injury-UP signature are also upregulated in the two AD bulk tissue RNA-seq datasets from ROSMAP and MSBB. In contrast, the mouse chronic neurodegenerative-UP and all the DOWN signatures were not reflected in these large AD datasets. Similarly, none of the three mouse astrocyte signatures was clearly present in the two astrocyte-specific AD datasets, nor could they be validated in the two human PD and the ALS datasets.

## Discussion

The main finding of this study is that, while sharing a common (pan-injury) astrocyte gene expression signature, mouse models of acute CNS injuries and neurodegenerative diseases are associated with very distinct astrocyte transcriptomic responses. On the one hand, astrocyte response to acute injury is remarkably characterized by an increased protein synthesis and degradation. Protein synthesis is unleashed by both upregulating the translational machinery of elongation factors (*Eef1e1*, *Eif2b2*, *Eif2b3*) and aminoacyl tRNA synthetases (*Cars*, *Iars*, *Kars*) and downregulating transcriptional repressors. Proteins involved in trafficking between the ER and the Golgi apparatus are also upregulated, likely reflecting the increasing demand for the folding, maturation, and intracellular transport of newly synthetized proteins. This massive protein synthesis is paralleled by an increased activity of protein degradation systems including the ubiquitin-proteasome system (i.e., upregulation of chaperones, ubiquitin ligases, and proteasome subunits) and autophagy (i.e., upregulation of lysosomal membrane proteins and enzymes), which probably serves the elimination of phagocytosed apoptotic and necrotic neurons from the injury area. In addition, the acute injury astrocyte signature consists of an immune response involving the upregulation of *Rela* (encoding for the NFκB effector subunit p65) and *Il33* (encoding for interleukin-33) and the downregulation of *Rcan2* (encoding for the regulator of calcineurin 2), among other genes. Of note, interleukin-33 is a nuclear alarmin expressed by astrocytes that induces microglial engulfment of synapses during development [55, 56], and is neuroprotective in multiple models of acute CNS injury by shifting microglial phenotype towards a phagocytic one [57–62]. Lastly, the acute injury signature included an upregulation of anti-oxidant defenses exemplified by both *Nfe2l2* (encoding the nuclear factor erythroid-derived 2-like 2 or NRF2), which is a major regulator of cellular anti-oxidant defenses and confers neuroprotection in acute CNS injuries [63–66] and neurodegenerative disease models [67, 68], and *Gss,* an NRF2 target gene encoding for the anti-oxidant enzyme glutathione synthetase.

In sharp contrast, the chronic reaction signature seen in neurodegenerative disease mouse models was much smaller, with the downregulated genes exceeding the number of upregulated ones. This could be partially explained by the fact that the median number of DEGs contributing to the meta-analysis was smaller for the chronic neurodegeneration datasets compared to acute injury datasets (UP, 823 vs 1402; DOWN, 775 vs 1468). An alternative explanation could be that our meta-analysis combined datasets obtained from very different models, CNS regions, and age groups, with disparate methodologies of astrocyte isolation or astrocyte RNA purification, and different transcriptomic methods (microarray versus RNA-seq). There are regional differences in mouse astrocyte transcriptome, and aging has been associated with region-specific transcriptomic changes in mouse astrocytes [69–72]. Moreover, technological heterogeneity could be another contributor: for example, RNA-seq has several advantages over microarrays such as improved sensitivity, wider dynamic range, and the ability to detect novel and non-coding transcripts. This heterogeneity could have limited our ability to identify a common signature across disease mouse models and may have had a greater impact in the meta-analysis of chronic neurodegeneration datasets than in the meta-analysis of acute injury datasets. However, inspection of Table 1 reveals a similar degree of heterogeneity across acute injury and chronic neurodegeneration mouse studies, which did not preclude obtaining a sizable acute injury signature after meta-analysis. Thus, the findings of fewer DEGs in the chronic neurodegeneration datasets and a smaller chronic neurodegeneration signature compared to the acute injury datasets and signature, respectively, support the idea that mouse reactive astrocytes mount a less vigorous response in chronic relative to acute injuries.

A closer inspection of the list of chronic neurodegeneration-UP genes revealed that three of these genes are secreted extracellular matrix proteins: *Cyr61* (CCN1 or cellular communication network factor 1), *Lama4* (laminin α4), and *Thbs4* (thrombospondin 4). Cyr61/CCN1 is a secreted matricellular protein that has been involved in cell-cell adhesion, angiogenesis, and arborization of dendrites of hippocampal neurons [73–76]. Immunohistochemical studies have revealed an upregulation of several laminins in reactive astrocytes in AD, Down’s syndrome, and ALS [77–79]. Thrombospondins are astrocyte-secreted matricellular proteins involved in synaptogenesis [80, 81]. Since Aβ peptide can lead to synapse loss by inhibiting the secretion of thrombospondin 1 (TSP-1) by astrocytes [82, 83], and the P301S tauopathy mouse model exhibits decreased levels of TSP-1 in the brain [84], this upregulation of *Thbs4* in neurodegenerative disease models could be compensatory to a decrease in TSP-1 levels. Three other transcripts upregulated in chronic neurodegeneration models encode for transcription factors: *Gatad2b*, *Nr4a3*, and *Tsc22d1*. Remarkably, GATA zinc finger domain containing 2B (GATAD2B) is a transcriptional repressor whose loss of function mutations have been associated with intellectual disability and synapse loss [85]. Nuclear receptor subfamily 4 group A member 3 has been involved in Lewy body disease and multiple system atrophy [86]. TSC22D1 is a pro-apoptotic tumor suppressor transcription factor induced by transforming growth factor β (TGFβ), that was found as putatively involved in AD pathophysiology in an unbiased network analysis of transcription factors and their targets [87] and was also present in our acute injury-DOWN signature. The presence of *App* (encoding the amyloid β precursor protein or AβPP) in the neurodegeneration-UP signature is intriguing and cannot just be explained by a leakage of the *APP* transgene expression to astrocytes in AβPP-overexpressing AD mouse models [88], because it was also notably upregulated in other mouse models such as SOD1-G93A ALS mice. Increased AβPP expression has been reported in reactive astrocytes under certain conditions, both in vitro [89] and in vivo [90–92].

Among the chronic neurodegeneration-DOWN genes, *Agl*, *S1pr1,* and *Sod2* stand out. α-Glycosidase (also called α-amylase), encoded by *Agl*, is the rate-limiting enzyme of glycogenolysis and its downregulation in neurodegenerative mouse models implies an impairment of the energy supply to neurons through the astrocyte-neuron lactate shuttle. However, while *Agl* mRNA levels were found to be reduced in the hippocampal formation of AD patients compared to non-demented controls, an increased AGL immunoreactivity has been reported in AD reactive astrocytes [93, 94]. The sphingosine phosphate receptors 1 and 3 (S1PR1 and S1PR3) are upregulated by astrocytes in MS lesions [95–97], and their modulation by the FDA-approved MS drug and sphingosine analog fingolimod has beneficial anti-inflammatory and neurotrophic effects not only in MS but also in AD mouse models [98–101]. Deficiency of the anti-oxidant enzyme superoxide dismutase 2 (SOD2) in astrocytes leads to astrocyte oxidative stress [102], and a recent single nuclei RNA-seq study has found a downregulation of *Sod2* in astrocytes from human AD brains [103]. On the other hand, post-mortem neuropathological studies have reported an enhanced SOD2 immunoreactivity in astrocytes in several neurodegenerative diseases such as ALS [104, 105] and FTLD-tau and TDP-43 [106], suggesting the existence of a mismatch either between mRNA and protein levels or between human disease and mouse models.

Besides these acute and chronic injury-specific signatures, our meta-analysis rendered a pan-injury signature with genes upregulated and downregulated in both types of CNS conditions. Our pan-injury-UP signature included 5 of the 13 genes proposed by Liddelow et al. as pan-reactive: *Cd44*, *Gfap*, *Osmr*, *Timp1*, and *Vim*. In addition, the putative A1 (neurotoxic) genes *C3* and *Serping1* were part of our pan-injury-UP signature [2]. Other pan-injury upregulated genes were *Lgals1*, *Lgals3,* and *Lgals3bp* encoding galectins 1 and 3 and galectin 3 binding protein, respectively. Astrocyte galectin-1 has been associated with worsening neurodegeneration of motor neurons in an ALS mouse model [107], but with neurotrophic and anti-inflammatory effects in models of MS [108] and brain ischemia [109, 110]. The pan-injury-DOWN signature included genes involved in lipid metabolism (*Acss2*, *Bbox1*, *Elovl2*, *Fzd2*, *Hmgcs1*, *Sqle*), and other relevant genes such as *Apln, Insig1*, *Ppargc1a,* and *Ttpa*. *Apln* encodes for apelin, a secreted peptide that has been shown to be neuroprotective in multiple acute injury and chronic neurodegenerative models [111–115]. *Insig1* encodes for insulin induced gene 1 and its downregulation suggests a globally reduced insulin signaling [116]. *Ppargc1a* encodes for the peroxisome proliferator activator receptor γ coactivator 1α, and it is noteworthy that PPARγ agonists have been proposed as anti-inflammatory drugs for many CNS conditions [117]. *Ttpa* encodes for α-tocopherol (vitamin E) transfer protein and, thus, has an anti-oxidant function; indeed, its deficiency leads to oxidative stress and enhanced Aβ deposition in transgenic AD mice [118, 119].

While BrDU incorporation experiments and immunohistochemistry for proliferative markers (i.e., Ki67) are better suited to examine astrocyte proliferation in mouse models of CNS injury, our GSEA of relevant gene sets suggests that pro-inflammatory signaling pathways (JAK/STAT, MAPK, NFκB, calcineurin/NFAT) are preponderant over proliferative signaling pathways (Wnt/β-catenin and Sonic hedgehog) in both acute and chronic conditions, supporting the idea that astrocyte reaction represents mainly a phenotypic change of existing astrocytes rather than proliferation of glial progenitors [8, 120].

Finally, our exploratory validation of these mouse astrocyte signatures in human neurodegenerative transcriptomic datasets revealed little concordance, arguing for caution when extrapolating the findings of astrocyte mouse transcriptomic studies to the human disease scenario. These mouse versus human discrepancies can be explained by a combination of reasons: (1) there are inherent genomic, transcriptomic, and lifespan/aging differences between mice and humans [121]; (2) mouse models are unlikely to recapitulate the high degree of heterogeneity and complexity of human diseases; (3) most human transcriptomic datasets available were obtained from bulk tissue, which by definition dilutes the astrocyte transcripts among those from all the other cell types and reduces the sensitivity to detect astrocyte-specific changes; (4) the isolation of astrocytes in the two human astrocyte-specific AD datasets was based on their GFAP immunoreactivity, likely biasing the astrocyte population towards a reactive phenotype in both AD and control individuals, and thus limiting the ability to find differences between both groups; and (5) there is a notable overlap in transcriptome between astrocytes and other cell types, singularly microglia. While recent single nuclei RNA-seq in AD and control human brains have produced quantitatively limited astrocyte datasets [103, 122, 123], future astrocyte-enriched single nuclei RNA-seq studies from various human diseases will warrant a similar methodological approach and allow a more definite validation of the present findings from mouse models.

## Conclusions

In summary, our meta-analysis of publicly available astrocyte transcriptomic datasets from multiple acute and chronic CNS injury mouse models highlights the heterogeneous nature of astrocyte reaction and provides important clues about its context dependence.

## Supplementary information

**Additional file 1: Figure S1.** Meta-analysis shows distinct acute injury and chronic neurodegeneration signatures, and a common pan-injury signature. Heatmaps depict − log_10_(p value) of up-regulated (UP) and down-regulated (DOWN) genes comprising the acute injury (AI), chronic neurodegeneration (ND) and pan-injury (PAN) signatures.

**Additional file 2: Figure S2.** Inflammatory but not proliferation signaling pathways are up-regulated in acute injury and chronic neurodegeneration. Heatmap shows the –log_10_(p value) of pro-inflammatory (NFκB, calcineurin/NFAT, MAPK, and JAK/STAT) and proliferative (Wnt/β-catenin and sonic hedgehog) gene sets in the astrocyte transcriptomic datasets analyzed, corresponding to the GSEA in Figure 7. The gene sets are color-coded by pathway and ordered by descending meta-analytic *p* value [− log10(Meta adj. p)]. Note that NFκB, MAPK and JAK/STAT gene sets are statistically more significant than Wnt/β-catenin and sonic hedgehog.

**Additional file 3: Figure S3.** Validation of mouse astrocyte transcriptomic signatures in human neurodegenerative transcriptomic datasets. Violin plots represent the logFC of all genes (both statistically significant and non-significant) from the acute injury (AI), chronic neurodegenerative (ND) and pan-injury (PAN) mouse astrocyte signatures in diseased versus control human subjects. Abbreviations: AD-DLPC = AD dorsolateral prefrontal cortex from ROSMAP; AD-PHG = AD parahippocampal gyrus from MSBB; AD-astro1 = GFAP+ astrocytes laser capture microdissected from lateral temporal cortex; AD-astro2 = GFAP+ astrocytes sorted from superior frontal cortex; ALS-SC = amyotrophic lateral sclerosis spinal cord gray matter; PD-SN = Parkinson’s disease *substantia nigra*; PD-Str = Parkinson’s disease *striatum*.

**Additional file 4: Table S1. **

## Data Availability

The datasets used and/or analyzed during the current study are available from the corresponding author on reasonable request.

## Abbreviations

Ab: Antibody
Aβ: Amyloid β peptide
AβPP: Amyloid-β precursor protein
AD: Alzheimer’s disease
AI: Acute injury
Aldh1L1: Aldehyde dehydrogenase 1 L1
AGL: α-Glycosidase
ALS: Amyotrophic lateral sclerosis
CA1: Cornus ammonis 1
CaN-NFAT: Calcineurin-nuclear factor activating of T-cells
cc: Corpus callosum
CCN1: Cellular communication network factor 1
CNS: Central nervous system
CTRL: Control
ctx: Cortex
Cx43: Connexin-43
DEGs: Differentially expressed genes
DLPC: Dorsolateral prefrontal cortex
DOWN: Downregulated
eGFP: Enhanced green fluorescent protein
ER: Endoplasmic reticulum
FACS: Fluorescence-activated cell sorting
FDA: Food and Drug Administration
FDR: False discovery rate
FKPM: Fragments per kilobase of transcript per million mapped reads
FTLD: Frontotemporal lobar degeneration
GATAD2B: GATA zinc finger domain containing 2B
GFAP: Glial fibrillary acidic protein
Glt1: Glutamate transporter 1
GO: Gene Ontology
GSEA: Gene set enrichment analysis
i.p.: Intra-peritoneal
JAK/STAT: Janus Kinases/Signal Transducer and Activator of Transcription
KEGG: Kyoto Encyclopedia of Genes and Genomes
LCM: Laser capture microdissection
logFC: Logarithm of fold-change
LPS: Lipopolysaccharide
MAPK: Mitogen-activated protein kinase
MAPT: Microtubule-associated protein tau
MCAO: Middle cerebral artery occlusion
MPTP: 1-Methyl-4-phenyl-1,2,3,6-tetrahydropyridine
MS: Multiple sclerosis
MSBB: Mount Sinai Brain Bank
ND: Neurodegeneration
NES: Normalized enrichment score
NFκB: Nuclear factor kappa B
NFT: Neurofibrillary tangle
NRF2: Nuclear factor erythroid-derived 2-like 2
PAN: Pan-injury
PD: Parkinson’s disease
PHG: Parahippocampal gyrus
PPARγ: Peroxisome proliferator activator receptor γ
PS1/2: Presenilin 1/2
RNA-seq: Ribonucleic acid sequencing
ROSMAP: Religious Orders Study and Memory and Aging Project
S1PR1/3: Sphingosine phosphate receptors 1/3
sac: Sacrifice
SALS: Sporadic ALS
SC: Spinal cord
SCI: Spinal cord injury
SN: Substantia nigra
SOD1/2: Superoxide dismutase 1/2
Str: Striatum
TBI: Traumatic brain injury
TDP-43: Tar DNA-binding protein 43 kDa
TGFβ: Transforming growth factor β
TRAP: Translating ribosome affinity purification
TSP-1: Thrombospondin 1
UP: Upregulated

## References

[1] Zamanian JL, Xu L, Foo LC, Nouri N, Zhou L, Giffard RG (2012). Genomic analysis of reactive astrogliosis. J Neurosci Off J Soc Neurosci..

[2] Liddelow SA, Guttenplan KA, Clarke LE, Bennett FC, Bohlen CJ, Schirmer L (2017). Neurotoxic reactive astrocytes are induced by activated microglia. Nature.

[3] Buffo A, Rite I, Tripathi P, Lepier A, Colak D, Horn A-P (2008). Origin and progeny of reactive gliosis: a source of multipotent cells in the injured brain. Proc Natl Acad Sci U S A..

[4] Bardehle S, Krüger M, Buggenthin F, Schwausch J, Ninkovic J, Clevers H (2013). Live imaging of astrocyte responses to acute injury reveals selective juxtavascular proliferation. Nat Neurosci..

[5] Sirko S, Behrendt G, Johansson PA, Tripathi P, Costa M, Bek S (2013). Reactive glia in the injured brain acquire stem cell properties in response to sonic hedgehog. [corrected]. Cell Stem Cell..

[6] Bondolfi L, Calhoun M, Ermini F, Kuhn HG, Wiederhold K-H, Walker L (2002). Amyloid-associated neuron loss and gliogenesis in the neocortex of amyloid precursor protein transgenic mice. J Neurosci Off J Soc Neurosci..

[7] Kamphuis W, Orre M, Kooijman L, Dahmen M, Hol EM (2012). Differential cell proliferation in the cortex of the APPswePS1dE9 Alzheimer’s disease mouse model. Glia..

[8] Serrano-Pozo A, Gómez-Isla T, Growdon JH, Frosch MP, Hyman BT (2013). A phenotypic change but not proliferation underlies glial responses in Alzheimer disease. Am J Pathol..

[9] Lepore AC, Dejea C, Carmen J, Rauck B, Kerr DA, Sofroniew MV (2008). Selective ablation of proliferating astrocytes does not affect disease outcome in either acute or chronic models of motor neuron degeneration. Exp Neurol..

[10] R Core Team (2018). R: a language and environment for statistical computing. R Found Stat Comput.

[11] Gene Expression Omnibus. [cited 2020 Jan 12]; Available from: https://www.ncbi.nlm.nih.gov/geo/.

[12] Rakers C, Schleif M, Blank N, Matušková H, Ulas T, Händler K (2019). Stroke target identification guided by astrocyte transcriptome analysis. Glia..

[13] Anderson MA, Burda JE, Ren Y, Ao Y, O’Shea TM, Kawaguchi R (2016). Astrocyte scar formation aids central nervous system axon regeneration. Nature..

[14] Noristani HN, Sabourin JC, Boukhaddaoui H, Chan-Seng E, Gerber YN, Perrin FE (2016). Spinal cord injury induces astroglial conversion towards neuronal lineage. Mol Neurodegener.

[15] Michalovicz LT, Kelly KA, Vashishtha S, Ben-Hamo R, Efroni S, Miller JV (2019). Astrocyte-specific transcriptome analysis using the ALDH1L1 bacTRAP mouse reveals novel biomarkers of astrogliosis in response to neurotoxicity. J Neurochem..

[16] Miller SJ, Glatzer JC, Hsieh Y-C, Rothstein JD (2018). Cortical astroglia undergo transcriptomic dysregulation in the G93A SOD1 ALS mouse model. J Neurogenet..

[17] Baker DJ, Blackburn DJ, Keatinge M, Sokhi D, Viskaitis P, Heath PR (2015). Lysosomal and phagocytic activity is increased in astrocytes during disease progression in the SOD1 (G93A) mouse model of amyotrophic lateral sclerosis. Front Cell Neurosci..

[18] Sun S, Sun Y, Ling S-C, Ferraiuolo L, McAlonis-Downes M, Zou Y (2015). Translational profiling identifies a cascade of damage initiated in motor neurons and spreading to glia in mutant SOD1-mediated ALS. Proc Natl Acad Sci U S A..

[19] Orre M, Kamphuis W, Osborn LM, Jansen AHP, Kooijman L, Bossers K (2014). Isolation of glia from Alzheimer’s mice reveals inflammation and dysfunction. Neurobiol Aging..

[20] Kamphuis W, Kooijman L, Orre M, Stassen O, Pekny M, Hol EM (2015). GFAP and vimentin deficiency alters gene expression in astrocytes and microglia in wild-type mice and changes the transcriptional response of reactive glia in mouse model for Alzheimer’s disease. Glia..

[21] Ceyzériat K, Ben Haim L, Denizot A, Pommier D, Matos M, Guillemaud O (2018). Modulation of astrocyte reactivity improves functional deficits in mouse models of Alzheimer’s disease. Acta Neuropathol Commun.

[22] Srinivasan K, Friedman BA, Larson JL, Lauffer BE, Goldstein LD, Appling LL (2016). Untangling the brain’s neuroinflammatory and neurodegenerative transcriptional responses. Nat Commun..

[23] Wu T, Dejanovic B, Gandham VD, Gogineni A, Edmonds R, Schauer S (2019). Complement C3 is activated in human AD brain and is required for neurodegeneration in mouse models of amyloidosis and tauopathy. Cell Rep.

[24] Patro R, Duggal G, Love MI, Irizarry RA, Kingsford C (2017). Salmon provides fast and bias-aware quantification of transcript expression. Nat Methods..

[25] Ritchie ME, Phipson B, Wu D, Hu Y, Law CW, Shi W (2015). limma powers differential expression analyses for RNA-sequencing and microarray studies. Nucleic Acids Res.

[26] Law CW, Chen Y, Shi W, Smyth GK (2014). voom: precision weights unlock linear model analysis tools for RNA-seq read counts. Genome Biol.

[27] Dewey M (2019). Metap: meta-analysis of significance values. R Package Version 11.

[28] Zhang Y, Chen K, Sloan SA, Bennett ML, Scholze AR, O’Keeffe S (2014). An RNA-sequencing transcriptome and splicing database of glia, neurons, and vascular cells of the cerebral cortex. J Neurosci Off J Soc Neurosci..

[29] Liberzon A, Birger C, Thorvaldsdóttir H, Ghandi M, Mesirov JP, Tamayo P (2015). The Molecular Signatures Database (MSigDB) hallmark gene set collection. Cell Syst..

[30] Subramanian A, Tamayo P, Mootha VK, Mukherjee S, Ebert BL, Gillette MA (2005). Gene set enrichment analysis: a knowledge-based approach for interpreting genome-wide expression profiles. Proc Natl Acad Sci U S A..

[31] Gene Set Enrichment Analysis. [cited 2019 Dec 17]; Available from: http://software.broadinstitute.org/gsea/index.jsp.

[32] De Jager PL, Ma Y, McCabe C, Xu J, Vardarajan BN, Felsky D (2018). A multi-omic atlas of the human frontal cortex for aging and Alzheimer’s disease research. Sci Data.

[33] Wang M, Beckmann ND, Roussos P, Wang E, Zhou X, Wang Q (2018). The Mount Sinai cohort of large-scale genomic, transcriptomic and proteomic data in Alzheimer’s disease. Sci Data.

[34] Simpson JE, Ince PG, Shaw PJ, Heath PR, Raman R, Garwood CJ (2011). Microarray analysis of the astrocyte transcriptome in the aging brain: relationship to Alzheimer’s pathology and APOE genotype. Neurobiol Aging..

[35] Riley BE, Gardai SJ, Emig-Agius D, Bessarabova M, Ivliev AE, Schüle B (2014). Systems-based analyses of brain regions functionally impacted in Parkinson’s disease reveals underlying causal mechanisms. PloS One..

[36] Dangond F, Hwang D, Camelo S, Pasinelli P, Frosch MP, Stephanopoulos G (2004). Molecular signature of late-stage human ALS revealed by expression profiling of postmortem spinal cord gray matter. Physiol Genomics..

[37] Bihlmeyer NA, Merrill E, Lambert Y, Srivastava GP, Clark TW, Hyman BT (2019). Novel methods for integration and visualization of genomics and genetics data in Alzheimer’s disease. Alzheimers Dement J Alzheimers Assoc..

[38] Wickham H (2016). ggplot2: elegant graphics for data analysis.

[39] Muñoz-Manchado AB, Villadiego J, Suárez-Luna N, Bermejo-Navas A, Garrido-Gil P, Labandeira-García JL (2013). Neuroprotective and reparative effects of carotid body grafts in a chronic MPTP model of Parkinson’s disease. Neurobiol Aging..

[40] Ouali Alami N, Schurr C, Olde Heuvel F, Tang L, Li Q, Tasdogan A (2018). NF-κB activation in astrocytes drives a stage-specific beneficial neuroimmunological response in ALS. EMBO J..

[41] Norris CM, Kadish I, Blalock EM, Chen K-C, Thibault V, Porter NM (2005). Calcineurin triggers reactive/inflammatory processes in astrocytes and is upregulated in aging and Alzheimer’s models. J Neurosci Off J Soc Neurosci..

[42] Abdul HM, Sama MA, Furman JL, Mathis DM, Beckett TL, Weidner AM (2009). Cognitive decline in Alzheimer’s disease is associated with selective changes in calcineurin/NFAT signaling. J Neurosci Off J Soc Neurosci..

[43] Furman JL, Sama DM, Gant JC, Beckett TL, Murphy MP, Bachstetter AD (2012). Targeting astrocytes ameliorates neurologic changes in a mouse model of Alzheimer’s disease. J Neurosci Off J Soc Neurosci..

[44] Sompol P, Furman JL, Pleiss MM, Kraner SD, Artiushin IA, Batten SR (2017). Calcineurin/NFAT signaling in activated astrocytes drives network hyperexcitability in Aβ-bearing mice. J Neurosci Off J Soc Neurosci.

[45] Sriram K, Benkovic SA, Hebert MA, Miller DB, O’Callaghan JP (2004). Induction of gp130-related cytokines and activation of JAK2/STAT3 pathway in astrocytes precedes up-regulation of glial fibrillary acidic protein in the 1-methyl-4-phenyl-1,2,3,6-tetrahydropyridine model of neurodegeneration: key signaling pathway for astrogliosis in vivo?. J Biol Chem..

[46] Ben Haim L, Ceyzériat K, Carrillo-de Sauvage MA, Aubry F, Auregan G, Guillermier M (2015). The JAK/STAT3 pathway is a common inducer of astrocyte reactivity in Alzheimer’s and Huntington’s diseases. J Neurosci Off J Soc Neurosci..

[47] Levine J, Kwon E, Paez P, Yan W, Czerwieniec G, Loo JA (2016). Traumatically injured astrocytes release a proteomic signature modulated by STAT3-dependent cell survival. Glia..

[48] Hyman BT, Elvhage TE, Reiter J (1994). Extracellular signal regulated kinases. Localization of protein and mRNA in the human hippocampal formation in Alzheimer’s disease. Am J Pathol..

[49] Mandell JW, VandenBerg SR (1999). ERK/MAP kinase is chronically activated in human reactive astrocytes. Neuroreport..

[50] Cole-Edwards KK, Musto AE, Bazan NG (2006). c-Jun N-terminal kinase activation responses induced by hippocampal kindling are mediated by reactive astrocytes. J Neurosci Off J Soc Neurosci..

[51] McCoy E, Sontheimer H (2010). MAPK induces AQP1 expression in astrocytes following injury. Glia..

[52] White BD, Nathe RJ, Maris DO, Nguyen NK, Goodson JM, Moon RT (2010). Beta-catenin signaling increases in proliferating NG2+ progenitors and astrocytes during post-traumatic gliogenesis in the adult brain. Stem Cells Dayt Ohio..

[53] L’Episcopo F, Tirolo C, Testa N, Caniglia S, Morale MC, Cossetti C (2011). Reactive astrocytes and Wnt/β-catenin signaling link nigrostriatal injury to repair in 1-methyl-4-phenyl-1,2,3,6-tetrahydropyridine model of Parkinson’s disease. Neurobiol Dis..

[54] Amankulor NM, Hambardzumyan D, Pyonteck SM, Becher OJ, Joyce JA, Holland EC (2009). Sonic hedgehog pathway activation is induced by acute brain injury and regulated by injury-related inflammation. J Neurosci Off J Soc Neurosci..

[55] Wicher G, Husic E, Nilsson G, Forsberg-Nilsson K (2013). Developmental expression of IL-33 in the mouse brain. Neurosci Lett..

[56] Vainchtein ID, Chin G, Cho FS, Kelley KW, Miller JG, Chien EC (2018). Astrocyte-derived interleukin-33 promotes microglial synapse engulfment and neural circuit development. Science.

[57] Gadani SP, Walsh JT, Smirnov I, Zheng J, Kipnis J (2015). The glia-derived alarmin IL-33 orchestrates the immune response and promotes recovery following CNS injury. Neuron..

[58] Chen H, Sun Y, Lai L, Wu H, Xiao Y, Ming B (2015). Interleukin-33 is released in spinal cord and suppresses experimental autoimmune encephalomyelitis in mice. Neuroscience..

[59] Pomeshchik Y, Kidin I, Korhonen P, Savchenko E, Jaronen M, Lehtonen S (2015). Interleukin-33 treatment reduces secondary injury and improves functional recovery after contusion spinal cord injury. Brain Behav Immun..

[60] Yang Y, Liu H, Zhang H, Ye Q, Wang J, Yang B (2017). ST2/IL-33-dependent microglial response limits acute ischemic brain injury. J Neurosci Off J Soc Neurosci.

[61] Gao Y, Luo C-L, Li L-L, Ye G-H, Gao C, Wang H-C (2017). IL-33 provides neuroprotection through suppressing apoptotic, autophagic and NF-κB-mediated inflammatory pathways in a rat model of recurrent neonatal seizure. Front Mol Neurosci..

[62] Chen Z, Xu N, Dai X, Zhao C, Wu X, Shankar S (2019). Interleukin-33 reduces neuronal damage and white matter injury via selective microglia M2 polarization after intracerebral hemorrhage in rats. Brain Res Bull..

[63] Chen P-C, Vargas MR, Pani AK, Smeyne RJ, Johnson DA, Kan YW (2009). Nrf2-mediated neuroprotection in the MPTP mouse model of Parkinson’s disease: critical role for the astrocyte. Proc Natl Acad Sci U S A..

[64] Bell KF, Al-Mubarak B, Fowler JH, Baxter PS, Gupta K, Tsujita T (2011). Mild oxidative stress activates Nrf2 in astrocytes, which contributes to neuroprotective ischemic preconditioning. Proc Natl Acad Sci U S A..

[65] Xu J, Huang G, Zhang K, Sun J, Xu T, Li R (2014). Nrf2 activation in astrocytes contributes to spinal cord ischemic tolerance induced by hyperbaric oxygen preconditioning. J Neurotrauma..

[66] Draheim T, Liessem A, Scheld M, Wilms F, Weißflog M, Denecke B (2016). Activation of the astrocytic Nrf2/ARE system ameliorates the formation of demyelinating lesions in a multiple sclerosis animal model. Glia..

[67] Vargas MR, Johnson DA, Sirkis DW, Messing A, Johnson JA (2008). Nrf2 activation in astrocytes protects against neurodegeneration in mouse models of familial amyotrophic lateral sclerosis. J Neurosci Off J Soc Neurosci..

[68] Lastres-Becker I, Ulusoy A, Innamorato NG, Sahin G, Rábano A, Kirik D (2012). α-Synuclein expression and Nrf2 deficiency cooperate to aggravate protein aggregation, neuronal death and inflammation in early-stage Parkinson’s disease. Hum Mol Genet..

[69] John Lin C-C, Yu K, Hatcher A, Huang T-W, Lee HK, Carlson J (2017). Identification of diverse astrocyte populations and their malignant analogs. Nat Neurosci..

[70] Chai H, Diaz-Castro B, Shigetomi E, Monte E, Octeau JC, Yu X (2017). Neural circuit-specialized astrocytes: transcriptomic, proteomic, morphological, and functional evidence. Neuron.

[71] Clarke LE, Liddelow SA, Chakraborty C, Münch AE, Heiman M, Barres BA (2018). Normal aging induces A1-like astrocyte reactivity. Proc Natl Acad Sci U S A.

[72] Boisvert MM, Erikson GA, Shokhirev MN, Allen NJ (2018). The aging astrocyte transcriptome from multiple regions of the mouse brain. Cell Rep.

[73] Malik AR, Urbanska M, Gozdz A, Swiech LJ, Nagalski A, Perycz M (2013). Cyr61, a matricellular protein, is needed for dendritic arborization of hippocampal neurons. J Biol Chem..

[74] Jones EV, Bouvier DS (2014). Astrocyte-secreted matricellular proteins in CNS remodelling during development and disease. Neural Plast..

[75] Malik AR, Liszewska E, Jaworski J (2015). Matricellular proteins of the Cyr61/CTGF/NOV (CCN) family and the nervous system. Front Cell Neurosci..

[76] Jayakumar AR, Apeksha A, Norenberg MD (2017). Role of matricellular proteins in disorders of the central nervous system. Neurochem Res..

[77] Murtomäki S, Risteli J, Risteli L, Koivisto UM, Johansson S, Liesi P (1992). Laminin and its neurite outgrowth-promoting domain in the brain in Alzheimer’s disease and Down’s syndrome patients. J Neurosci Res..

[78] Palu E, Liesi P (2002). Differential distribution of laminins in Alzheimer disease and normal human brain tissue. J Neurosci Res..

[79] Wiksten M, Väänänen A, Liesi P (2007). Selective overexpression of gamma1 laminin in astrocytes in amyotrophic lateral sclerosis indicates an involvement in ALS pathology. J Neurosci Res..

[80] Christopherson KS, Ullian EM, Stokes CCA, Mullowney CE, Hell JW, Agah A (2005). Thrombospondins are astrocyte-secreted proteins that promote CNS synaptogenesis. Cell..

[81] Eroglu C, Allen NJ, Susman MW, O’Rourke NA, Park CY, Ozkan E (2009). Gabapentin receptor alpha2delta-1 is a neuronal thrombospondin receptor responsible for excitatory CNS synaptogenesis. Cell..

[82] Rama Rao KV, Curtis KM, Johnstone JT, Norenberg MD (2013). Amyloid-β inhibits thrombospondin 1 release from cultured astrocytes: effects on synaptic protein expression. J Neuropathol Exp Neurol..

[83] Son SM, Nam DW, Cha M-Y, Kim KH, Byun J, Ryu H (2015). Thrombospondin-1 prevents amyloid beta-mediated synaptic pathology in Alzheimer’s disease. Neurobiol Aging..

[84] Sidoryk-Wegrzynowicz M, Gerber YN, Ries M, Sastre M, Tolkovsky AM, Spillantini MG (2017). Astrocytes in mouse models of tauopathies acquire early deficits and lose neurosupportive functions. Acta Neuropathol Commun..

[85] Willemsen MH, Nijhof B, Fenckova M, Nillesen WM, Bongers EMHF, Castells-Nobau A (2013). GATAD2B loss-of-function mutations cause a recognisable syndrome with intellectual disability and are associated with learning deficits and synaptic undergrowth in Drosophila. J Med Genet..

[86] Kon T, Miki Y, Tanji K, Mori F, Tomiyama M, Toyoshima Y (2015). Localization of nuclear receptor subfamily 4, group A, member 3 (NR4A3) in Lewy body disease and multiple system atrophy. Neuropathol Off J Jpn Soc Neuropathol..

[87] Vargas DM, De Bastiani MA, Zimmer ER, Klamt F (2018). Alzheimer’s disease master regulators analysis: search for potential molecular targets and drug repositioning candidates. Alzheimers Res Ther.

[88] Heiland T, Zeitschel U, Puchades MA, Kuhn P-H, Lichtenthaler SF, Bjaalie JG (2019). Defined astrocytic expression of human amyloid precursor protein in Tg2576 mouse brain. Glia..

[89] Avila-Muñoz E, Arias C (2015). Cholesterol-induced astrocyte activation is associated with increased amyloid precursor protein expression and processing. Glia..

[90] Siman R, Card JP, Nelson RB, Davis LG (1989). Expression of beta-amyloid precursor protein in reactive astrocytes following neuronal damage. Neuron..

[91] Rohan de Silva HA, Jen A, Wickenden C, Jen LS, Wilkinson SL, Patel AJ (1997). Cell-specific expression of beta-amyloid precursor protein isoform mRNAs and proteins in neurons and astrocytes. Brain Res Mol Brain Res..

[92] Matsui T, Ingelsson M, Fukumoto H, Ramasamy K, Kowa H, Frosch MP (2007). Expression of APP pathway mRNAs and proteins in Alzheimer’s disease. Brain Res..

[93] Byman E, Schultz N (2018). Netherlands Brain Bank, Fex M, Wennström M. Brain alpha-amylase: a novel energy regulator important in Alzheimer disease?. Brain Pathol Zurich Switz..

[94] Byman E, Schultz N (2019). Netherlands Brain Bank, Blom AM, Wennström M. A potential role for α-amylase in amyloid-β-induced astrocytic glycogenolysis and activation. J Alzheimers Dis JAD..

[95] Nishimura H, Akiyama T, Irei I, Hamazaki S, Sadahira Y (2010). Cellular localization of sphingosine-1-phosphate receptor 1 expression in the human central nervous system. J Histochem Cytochem Off J Histochem Soc..

[96] Van Doorn R, Van Horssen J, Verzijl D, Witte M, Ronken E, Van Het Hof B (2010). Sphingosine 1-phosphate receptor 1 and 3 are upregulated in multiple sclerosis lesions. Glia..

[97] Brana C, Frossard MJ, Pescini Gobert R, Martinier N, Boschert U, Seabrook TJ (2014). Immunohistochemical detection of sphingosine-1-phosphate receptor 1 and 5 in human multiple sclerosis lesions. Neuropathol Appl Neurobiol..

[98] Fukumoto K, Mizoguchi H, Takeuchi H, Horiuchi H, Kawanokuchi J, Jin S (2014). Fingolimod increases brain-derived neurotrophic factor levels and ameliorates amyloid β-induced memory impairment. Behav Brain Res..

[99] Hoffmann FS, Hofereiter J, Rübsamen H, Melms J, Schwarz S, Faber H (2015). Fingolimod induces neuroprotective factors in human astrocytes. J Neuroinflammation..

[100] Aytan N, Choi J-K, Carreras I, Brinkmann V, Kowall NW, Jenkins BG (2016). Fingolimod modulates multiple neuroinflammatory markers in a mouse model of Alzheimer’s disease. Sci Rep.

[101] Carreras I, Aytan N, Choi J-K, Tognoni CM, Kowall NW, Jenkins BG (2019). Dual dose-dependent effects of fingolimod in a mouse model of Alzheimer’s disease. Sci Rep..

[102] Lee H-P, Pancholi N, Esposito L, Previll LA, Wang X, Zhu X (2012). Early induction of oxidative stress in mouse model of Alzheimer disease with reduced mitochondrial superoxide dismutase activity. PloS One..

[103] Zhou Y, Song WM, Andhey PS, Swain A, Levy T, Miller KR (2020). Human and mouse single-nucleus transcriptomics reveal TREM2-dependent and TREM2-independent cellular responses in Alzheimer’s disease. Nat Med..

[104] Shibata N, Asayama K, Hirano A, Kobayashi M (1996). Immunohistochemical study on superoxide dismutases in spinal cords from autopsied patients with amyotrophic lateral sclerosis. Dev Neurosci..

[105] Blaauwgeers HG (1996). Vianney de Jong JM, Verspaget HW, van den Berg FM, Troost D. Enhanced superoxide dismutase-2 immunoreactivity of astrocytes and occasional neurons in amyotrophic lateral sclerosis. J Neurol Sci..

[106] Martínez A, Carmona M, Portero-Otin M, Naudí A, Pamplona R, Ferrer I (2008). Type-dependent oxidative damage in frontotemporal lobar degeneration: cortical astrocytes are targets of oxidative damage. J Neuropathol Exp Neurol..

[107] Kobayakawa Y, Sakumi K, Kajitani K, Kadoya T, Horie H, Kira J-I (2015). Galectin-1 deficiency improves axonal swelling of motor neurones in SOD1(G93A) transgenic mice. Neuropathol Appl Neurobiol..

[108] Starossom SC, Mascanfroni ID, Imitola J, Cao L, Raddassi K, Hernandez SF (2012). Galectin-1 deactivates classically activated microglia and protects from inflammation-induced neurodegeneration. Immunity..

[109] Qu W, Wang Y, Wang J, Tang Y, Zhang Q, Tian D (2010). Galectin-1 enhances astrocytic BDNF production and improves functional outcome in rats following ischemia. Neurochem Res..

[110] Qu W-S, Wang Y-H, Ma J-F, Tian D-S, Zhang Q, Pan D-J (2011). Galectin-1 attenuates astrogliosis-associated injuries and improves recovery of rats following focal cerebral ischemia. J Neurochem..

[111] Kasai A, Kinjo T, Ishihara R, Sakai I, Ishimaru Y, Yoshioka Y (2011). Apelin deficiency accelerates the progression of amyotrophic lateral sclerosis. PloS One..

[112] Bao H-J, Zhang L, Han W-C, Dai D-K (2015). Apelin-13 attenuates traumatic brain injury-induced damage by suppressing autophagy. Neurochem Res..

[113] Bao H, Yang X, Huang Y, Qiu H, Huang G, Xiao H (2016). The neuroprotective effect of apelin-13 in a mouse model of intracerebral hemorrhage. Neurosci Lett.

[114] Duan J, Cui J, Yang Z, Guo C, Cao J, Xi M (2019). Neuroprotective effect of Apelin 13 on ischemic stroke by activating AMPK/GSK-3β/Nrf2 signaling. J Neuroinflammation..

[115] Zhu J, Dou S, Jiang Y, Chen J, Wang C, Cheng B (2019). Apelin-13 protects dopaminergic neurons in MPTP-induced Parkinson’s disease model mice through inhibiting endoplasmic reticulum stress and promoting autophagy. Brain Res..

[116] Arnold SE, Arvanitakis Z, Macauley-Rambach SL, Koenig AM, Wang H-Y, Ahima RS (2018). Brain insulin resistance in type 2 diabetes and Alzheimer disease: concepts and conundrums. Nat Rev Neurol..

[117] Heneka MT, Landreth GE, Hüll M (2007). Drug insight: effects mediated by peroxisome proliferator-activated receptor-gamma in CNS disorders. Nat Clin Pract Neurol..

[118] Nishida Y, Yokota T, Takahashi T, Uchihara T, Jishage K, Mizusawa H (2006). Deletion of vitamin E enhances phenotype of Alzheimer disease model mouse. Biochem Biophys Res Commun..

[119] Nishida Y, Ito S, Ohtsuki S, Yamamoto N, Takahashi T, Iwata N (2009). Depletion of vitamin E increases amyloid beta accumulation by decreasing its clearances from brain and blood in a mouse model of Alzheimer disease. J Biol Chem..

[120] Perez-Nievas BG, Serrano-Pozo A (2018). Deciphering the astrocyte reaction in Alzheimer’s disease. Front Aging Neurosci..

[121] Zhang Y, Sloan SA, Clarke LE, Caneda C, Plaza CA, Blumenthal PD (2016). Purification and characterization of progenitor and mature human astrocytes reveals transcriptional and functional differences with mouse. Neuron..

[122] Mathys H, Davila-Velderrain J, Peng Z, Gao F, Mohammadi S, Young JZ (2019). Single-cell transcriptomic analysis of Alzheimer’s disease. Nature..

[123] Grubman A, Chew G, Ouyang JF, Sun G, Choo XY, McLean C (2019). A single-cell atlas of entorhinal cortex from individuals with Alzheimer’s disease reveals cell-type-specific gene expression regulation. Nat Neurosci..

